# Supplementary material for: A Human Retinal Pigment Epithelium-Based Screening Platform Reveals Inducers of Photoreceptor Outer Segments Phagocytosis
Source: Stem Cell Reports. 2020 Nov 25;15(6):1347–61. doi: 10.1016/j.stemcr.2020.10.013 (PMC7724476; doi:10.1016/j.stemcr.2020.10.013)
Supplement: Document S1. Supplemental Experimental Procedures, Figures S1–S4, Tables S2, S4, S5, and S8 [file mmc1.pdf]

Stem Cell Reports, Volume *15*

Supplemental Information

A Human Retinal Pigment Epithelium-Based Screening Platform Reveals Inducers of Photoreceptor Outer Segments Phagocytosis

Sven Schreiter, Katerina Vafia, Rico Barsacchi, Stephen H. Tsang, Marc Bickle, Marius Ader, Mike O. Karl, Elly M. Tanaka, and Seba Almedawar

Supplemental Information: Figures and Legends

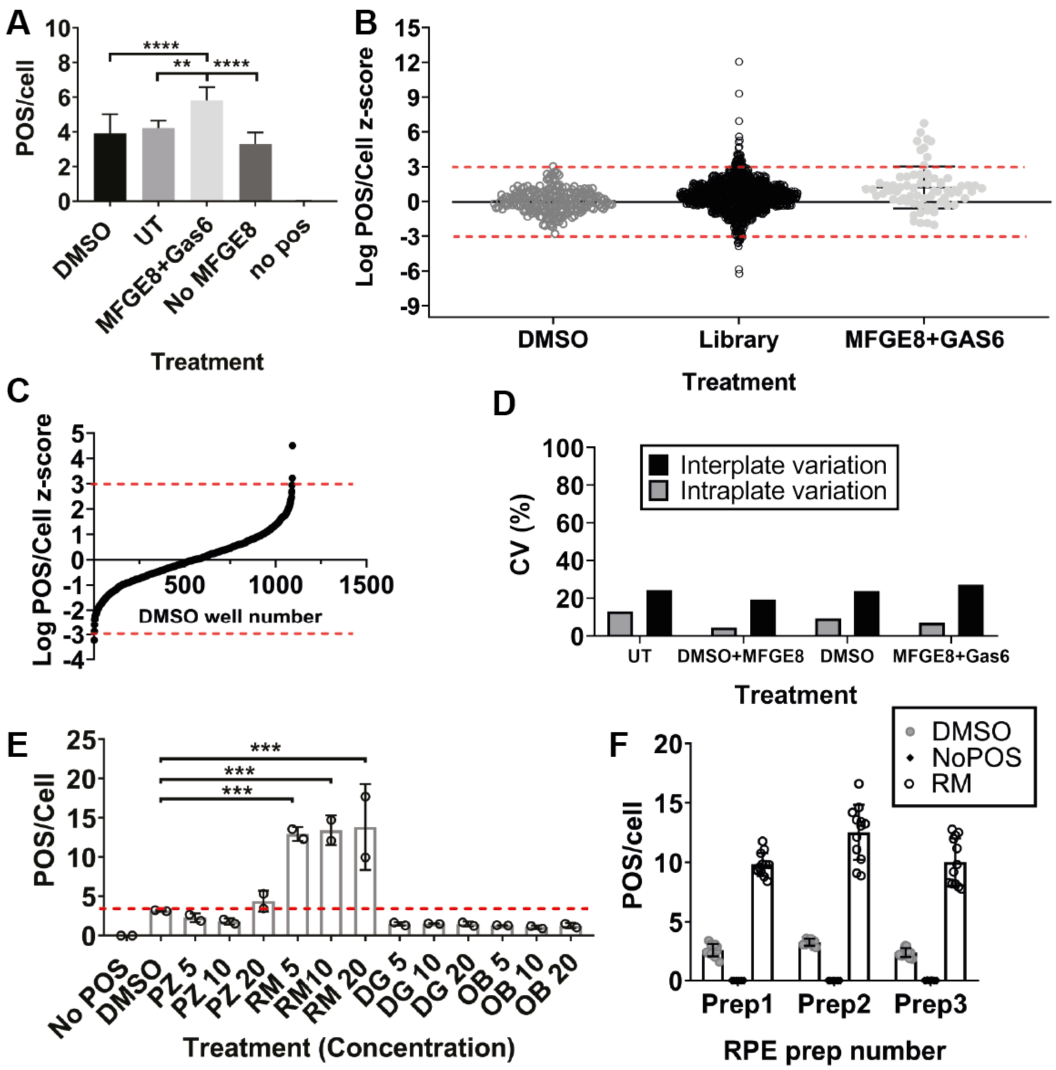

Figure S1. The screening platform demonstrates low false positive/negative rate and reproducibility across different human RPE preps. Related to Figure 3.

(A) Controls from one screening plate. MFGE8/GAS6: 2.5 µg/ml were added from each ligand. UT: Untreated. Data are represented as the mean ± SD. n=24 wells DMSO and n=4 wells of each treatment. Significance was calculated using one-way-ANOVA. All treatments were compared to DMSO. ns >0.05, *<0.05, ** <0.01, ***<0.001, ****<0.0001.

(B) The z-score of the log of the POS/cell within each DMSO, Library, or MFGE8+GAS6 well that was used in the screen was plotted. Dotted lines show the z-score threshold (-3, +3) that was used in the screen to determine hits. The graph shows that the library is spread over the z-score axis, while DMSO wells are concentrated between -3 and +3, and most of the MFGE8+GAS6 wells show a positive z-score.

(C) The z-score of the log of the POS/cell within each DMSO well that was used in the screen was plotted. The graph shows two wells that were identified as false positive and 1 that was identified as false negative.

(D) Interplate (all screening plates from 4 runs) and Intraplate (plate 1 from run 1) variation of the control wells used in the screen.

(E) Confirmation in transwells (Step III). Related to **Table S5**. Data are represented as the mean ± SD. N=2 biological repeats. Significance was calculated using one-way-ANOVA. All treatments were compared to DMSO. ns >0.05, *<0.05, ** <0.01, ***<0.001, ****<0.0001.

(F) Increase of POS phagocytosis using RM (RM) compared to DMSO was confirmed over 3 RPE differentiation rounds performed on different days. Data represent the mean of the total POS/cell count ± SD. n=12 wells per RPE differentiation batch.

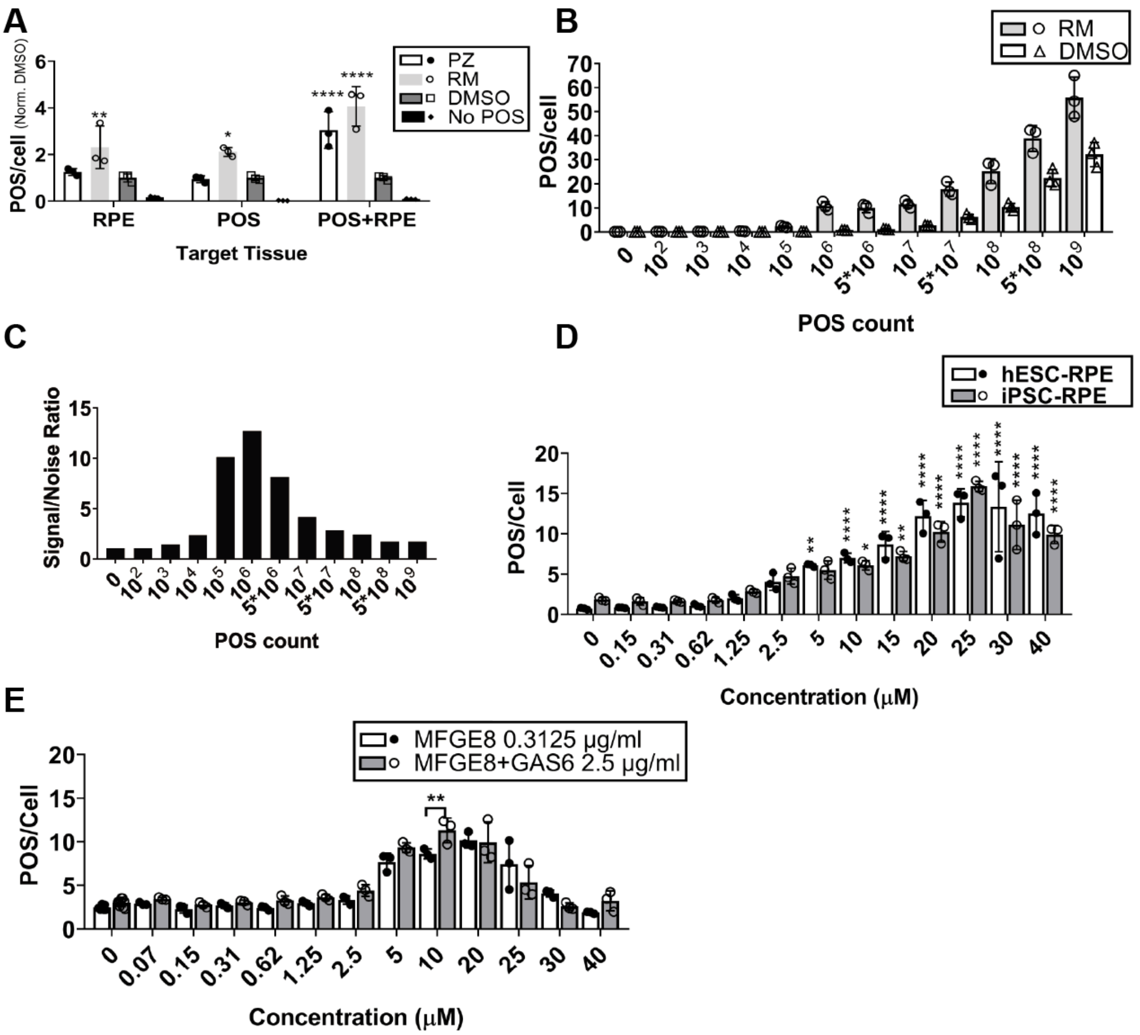

Figure S2. Orthogonal assays show that Ramoplanin acts on both photoreceptor and human RPE cells in a dose dependent manner. Related to Figure 3.

(A) Ramoplanin (RM)/Pirithione zinc (PZ)/ DMSO were added to hESC-RPE for 1 hour then they were washed off before addition of POS (Target Tissue: RPE), or added to AF555-labeled POS for 1 hour and washed off before addition of POS to RPE (Target Tissue: POS). Alternatively, AF555-labeled POS were added to the cells which were previously treated with the compounds without a washing step in between (Target Tissue: POS+RPE). RM seems to act on both POS and RPE. Data are normalized to DMSO in each target tissue separately and are represented as the mean ± SD. N=3 RPE differentiation batches. Significance was calculated using two-way-ANOVA. Samples of the same target tissue were compared to DMSO. ns >0.05, *<0.05, ** <0.01, ***<0.001, ****<0.0001.

(B) RM was added to RPE cells in the presence of different concentrations of AF555-labeled POS for 3 hours. Fixed cells were analyzed with confocal fluorescence microscopy. Addition of higher concentrations led to increase in the POS/cell count in both RM and DMSO. Data are represented as the mean ± SD. N=3 RPE differentiation batches.

(C) Ratio of the POS/cell count in RM treated samples to DMSO samples. The best signal to noise ratio was obtained when cells were challenged with 10^6 POS/well.

(D) Increasing concentrations of RM were added to RPE cells differentiated from hESC and iPSC in the presence of AF555-labeled POS for 3 hours. Fixed cells were analyzed with confocal fluorescence microscopy. Higher concentrations of RM increase the POS/cell count up to 25 µM. Addition of higher concentrations causes a drop in the POS/cell count. Data are represented as the mean ± SD. N=3 RPE differentiation batches. Significance was calculated using two-way-ANOVA. All treatments were compared to DMSO. ns >0.05, *<0.05, ** <0.01, ***<0.001, ****<0.0001.

(E) Increasing concentrations of RM were added to hESC-RPE in the presence of AF555-labeled POS and low MFGE8 concentration (0.3125 µg/ml) or MFGE8 and GAS6 (each at 2.5 µg/ml) for 3 hours. An additive effect is seen and is significant at 10 µM concentration of RM. Data are represented as the mean ± SD. N=3 RPE differentiation batches. Significance was calculated using two-way-ANOVA. Samples of the same RM concentration, but different ligands concentration were compared. ns >0.05, *<0.05, ** <0.01, ***<0.001, ****<0.0001.

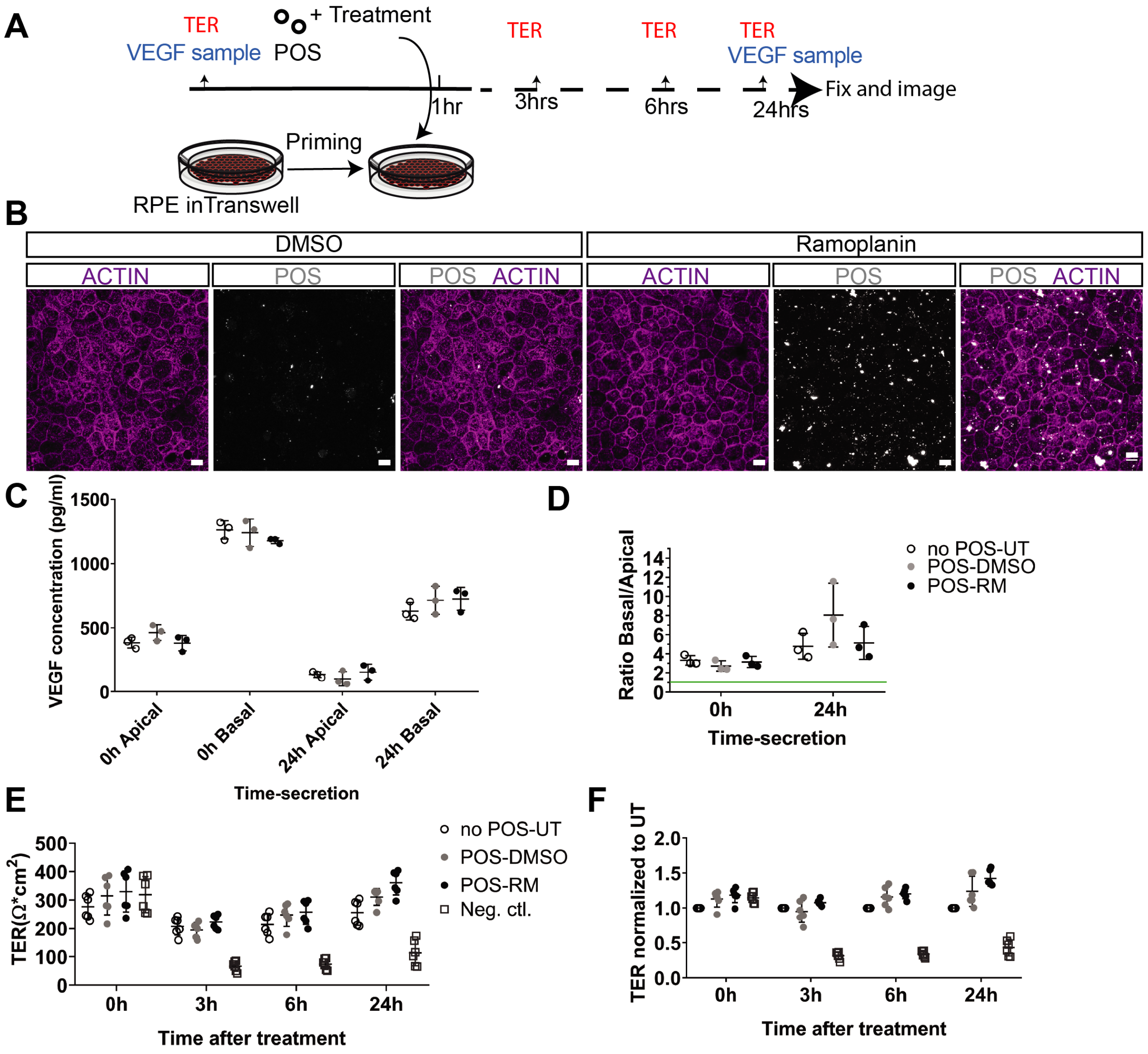

Figure S3. Ramoplanin does not affect human RPE polarized secretion of VEGF and RPE monolayer integrity. Related to Figure 4.

(A) Experiment pipeline.

(B) Fluorescence images of hESC-RPE cells cultured in transwells challenged with AF555-labeled POS (Gray) and Ramoplanin (RM)/DMSO for 3 hours. Fixed cells were labeled with Phalloidin (Magenta) to mark the boarders of the cells. Scale bar: 10 µm.

(C) VEGF values before and 24 hours after treatment with RM/DMSO and POS. Data are represented as the mean ± SD. N=3 RPE differentiation batches. RM does not compromise the polarized secretion of VEGF after 24 hours of treatment. UT: untreated.

(D) Ratio of the concentration of VEGF in the basal side of the RPE cells to the apical side. Data are represented as the mean ± SD. N=3 RPE differentiation batches.

(E) Transepithelial resistance (TER) values of RPE cells before and 3, 6, 24 hours after treatment with RM/DMSO and POS. A scratch was applied in the middle of the transwell to disturb the monolayer integrity (Neg. ctl.). Data are represented as the mean ± SD. N=3 RPE differentiation batches. RM does not compromise the TER of the cells after 24 hours of treatment.

(F) TER values were normalized to DMSO at each time point and plotted. Data are represented as the mean ± SD. N=3 RPE differentiation batches.

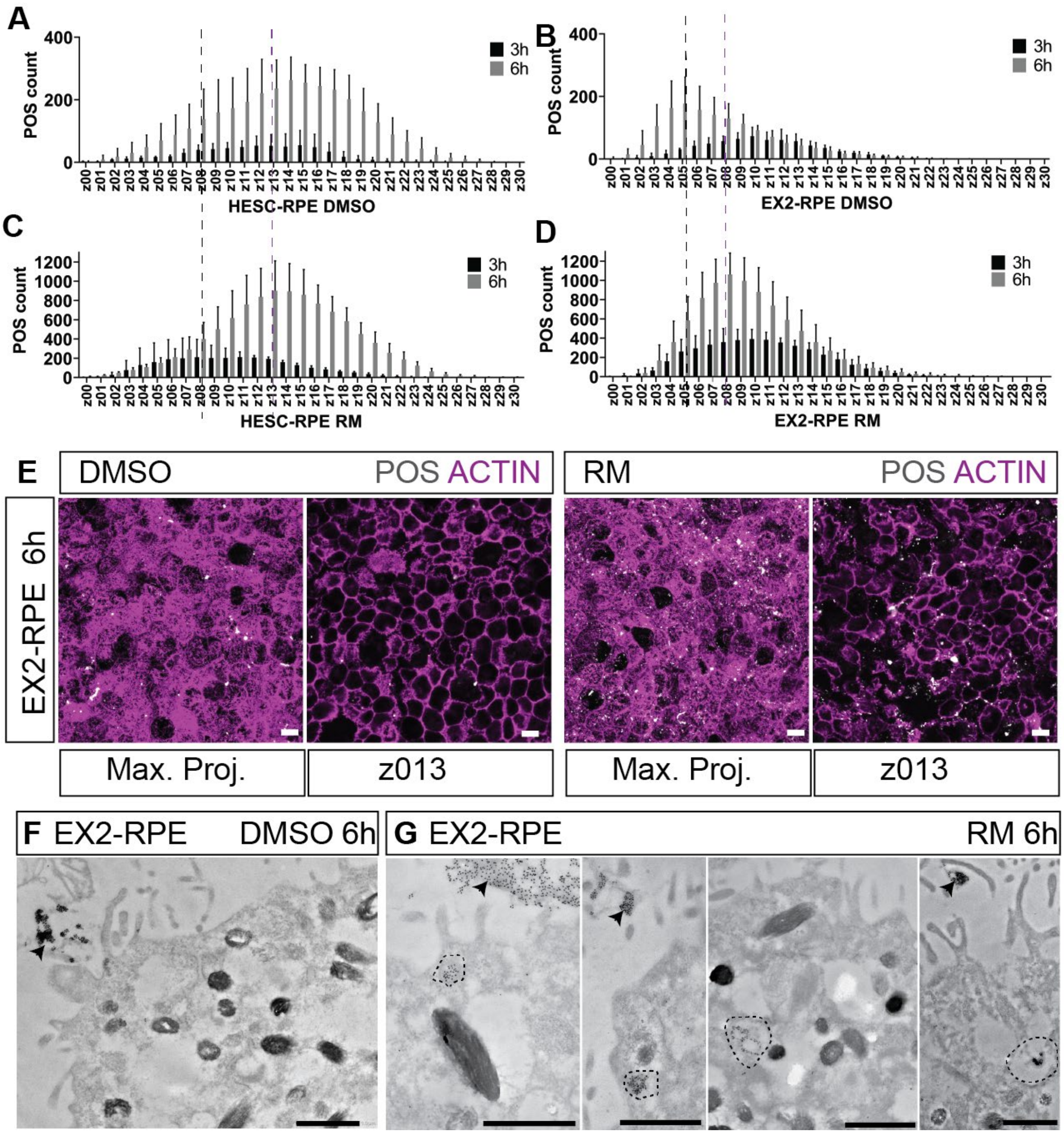

Figure S4. Ramoplanin rescues internalization defect in human MERTK mutant RPE determined by means of fluorescence confocal microscopy and transmission electron microscopy (TEM). Related to Figure 7.

(A-D) AF555- labeled POS were added to wild-type RPE (hESC-RPE) and MERTK mutant RPE (EX2-RPE) and left for 3 hours and 6 hours after POS addition. Cells were washed, fixed and labeled with phalloidin to mark the apical and the basal side of the cells. Confocal fluorescence microscopy images were acquired with 31 optical sections (z-stacks) and analyzed to obtain POS count. z0 is at the apical side of the RPE z30 is at the basal side of the RPE. Data represent the mean of the total POS count ± SD. N=3 RPE differentiation batches. In hESC-RPE POS are internalized after 6 hours and are mostly found in z13 (A). The amount of POS in z13 increases after treatment with Ramoplanin (RM) (C). At 6 hours POS still accumulate at the surface of the Ex2-RPE (z05, black dotted line, B), while in the presence of RM POS move towards the inside of the cell (z08, purple dotted line, D).

(E) Fluorescence microscopy images of EX2-RPE at 6 hours after addition of AF555-labeled POS and RM/DMSO as in **(B-D)**. Actin is labeled with phalloidin (Magenta). Maximum intensity projection of the 31 optical sections and z13 only are shown. The number of internalized POS is higher in z13 in RM treated samples compared to DMSO.

(F-G) Transmission electron microscopy of EX2-RPE cells treated with POS and DMSO/RM for 6 hours. In DMSO samples, POS are mostly found on the surface of the RPE, while in RM samples internalized POS at the apical side of the RPE could be detected. Scale bar: 1 μm.

Table S1. List of compounds in the FDA approved library (Excel Table). Related to Figure 3.

Table S2. List of hits from the primary screening assay. Related to Figure 3.

Compound name	Mean POS/cell DMSO	SD of the Mean POS/cell DMSO	POS/cell Hit	Z-score	Structure
CARBIDOPA	7.72627946	1.80183412	18.18239142	3.859843239	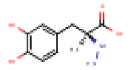
	3.97027337	0.72160097	6.987130292	3.293712615	
RAMOPLANIN	4.00344939	0.77791355	10.65762809	4.845892949	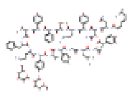
	4.31512749	0.62271494	8.368666042	9.050912693	
	6.1640321	1.53512574	19.56056543	4.942319314	
	3.536627	0.44530994	10.72073678	5.00557346	
CEPHAPIRIN SODIUM	4.02359632	0.61315362	6.35266788	3.007959189	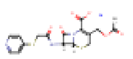
	6.24270555	1.32986439	13.73751677	3.388429658	
DOXORUBICIN	5.59807957	1.23901679	27.5891581	3.388646968	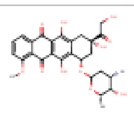
	4.73843874	1.26001387	11.65284639	7.655831648	
HOMIDIUM BROMIDE	3.3279917	0.66323947	39.13526909	13.12391821	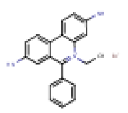
	3.38773503	0.74357468	53.79315086	11.13684767	
	4.42129613	0.74605771	96.48429397	18.78772985	
	6.69223467	1.3533011	110.1212104	14.40519773	
MERBROMIN	3.3279917	0.66323947	9.605061573	5.697155474	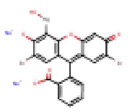
	3.38773503	0.74357468	16.28800234	6.371631183	
	10.8106779	1.49126386	16.77847338	3.185274372	
	6.69223467	1.3533011	15.69051785	4.449296127	
PYRITHIONE ZINC	4.42129613	0.74605771	8.062179186	3.725484414	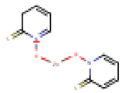
	10.8106779	1.49126386	20.57312327	4.631893696	
	6.69223467	1.3533011	18.92793233	5.407738765	
SUCCINYLSULFATHIAZOLE	1.76719904	0.19447114	2.795	4.406777297	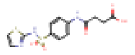
	7.18489185	1.19956523	12.08333333	3.286567331	
DIGOXIN	4.02359632	0.61315362	1.969020291	-4.518464121	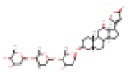
	6.24270555	1.32986439	2.655620834	-3.452450781	
OUABAIN	6.29444936	1.21391753	3.408987603	-3.68263515	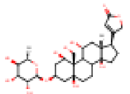
	5.93189068	1.51873745	2.351151505	-3.152237244	
POTASSIUM p-AMINOBENZOATE	4.31512749	0.62271494	1.842309897	-5.477229687	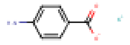
	4.00344939	0.77791355	1.270037248	-6.755468742	
	3.536627	0.44530994	1.525836523	-6.196451042	
SENNOSIDE A	3.50911	0.69084309	1.142751273	-5.504344515	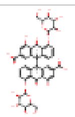
	6.42701984	1.32842536	2.999972133	-3.434578951	

Table S3. Autofluorescence values of hits in the absence of POS (Excel Table). Related to Figure 3.

Table S4. List of hits from confirmation I in 384-wells plates. Related to Figure 3.

Treatments	POS per Cell Mean	POS per Cell (Standard deviation)	z-score
**DMSO 0.2%**	8.150224049	1.931809451	
**No POS**	0.010885128	0.003674366	
**Digoxin 5**	4.419513352	0.934296173	-2.59094
**Digoxin 10**	4.536797936	1.368324695	-2.76922
**Digoxin 20**	5.18449646	0.988038768	-1.76115
**Ouabain 5**	4.594461362	1.3591871	-2.44608
**Ouabain 10**	4.509466427	1.841329131	-2.68072
**Ouabain 20**	5.62181552	0.988865715	-1.37035
**Proscillaridin 5**	4.700784182	1.53164408	-2.77648
**Proscillaridin 10**	4.725888169	1.694488286	-2.61604
**Proscillaridin 20**	5.453510783	0.950739994	-1.58906
**Pyrithione Zinc 5**	7.770393878	1.628158803	-0.27707
**Pyrithione Zinc 10**	8.762359994	2.009086981	0.490385
**Pyrithione Zinc 20**	20.89329247	1.723136342	4.360371
**Ramoplanin 5**	16.75894992	3.420217172	3.222757
**Ramoplanin 10**	19.17718295	2.73164563	4.030754
**Ramoplanin 20**	23.35658231	5.046969091	4.826715
**Thiostrepton 5**	8.94419779	1.348363893	0.46252
**Thiostrepton 10**	8.910353091	1.231485311	1.458945
**Thiostrepton 20**	10.82155098	2.236051978	1.429046

Table S5. List of hits from confirmation II in transwells. Related to Figure S1.

Treatment	POS/Cell	Median between fields	Mean between wells
**DMSO**	3.08	3.114	3.189
	5.168		
	3.148		
	2.212		
	3.272	3.264	
	3.66		
	3.096		
	3.256		
**Ouabain 5**	1.372	1.332	1.306
	1.2		
	1.292		
	1.66		
	1.292	1.28	
	1.4		
	1.268		
	1.164		
**Ouabain 10**	0.6	0.914	1.068
	1.084		
	0.744		
	1.172		
	2.504	1.222	
	1.304		
	1.14		
	0.852		
	1.416	1.508	1.266

**Ouabain 20**	1.468		
	1.872		
	1.548		
	1.016	1.024	
	0.96		
	1.324		
	1.032		
**Pyrithione Zinc 5**	2.872	2.684	2.284
	2.496		
	3.184		
	1.724		
	1.568	1.884	
	2.084		
	2.008		
	1.76		
**Pyrithione Zinc 10**	1.864	2.076	1.801
	4.116		
	2.288		
	1.448		
	1.756	1.526	
	1.728		
	1.208		
	1.324		
**Pyrithione Zinc 20**	3.368	3.448	4.396
	5.044		
	3.528		
	2.548		
	4.66	5.344	
	6.028		
	4.38		
	7.476		
**Ramoplanin 5**	9.392	13.564	12.939
	15.204		
	13.804		
	13.324		
	13.576	12.314	
	11.772		
	12.856		
	8.92		
**Ramoplanin 10**	16.588	14.744	13.415
	8.316		
	14.976		
	14.512		
	12.284	12.086	
	11.888		
	9.584		
	14.552		

**Ramoplanin 20**	8.268	9.966	13.843
	11.664		
	4.772		
	13.04		
	25.848	17.72	
	22.092		
	9.38		
	13.348		
**Digoxin 5**	1.4	1.312	1.484
	1.244		
	1.344		
	1.28		
	2.432	1.656	
	1.224		
	1.248		
	2.064		
**Digoxin 10**	1.724	1.548	1.523
	1.428		
	1.184		
	1.668		
	0.968	1.498	
	0.86		
	2.028		
	2.048		
**Digoxin 20**	2.12	1.684	1.464
	2.684		
	1.248		
	0.948		
	0.86	1.244	
	1.508		
	0.98		
	1.568		
**No POS**	0	0	0
	0		
	0		
	0		
	0	0	
	0.004		
	0		
	0		

Table S6. RNA seq data of samples treated with POS and Ramoplanin compared to samples treated with POS and DMSO (Excel Table). Related To Figure 5.

Table S7. IPA based analysis of RNA seq data (Excel Table). Related to Figure 5.

Table S8. Resources Table

Reagent or Resource	SOURCE	IDENTIFIER
**Antibodies**		
rhodopsin (TEM)	Sigma	O4886
rhodopsin (Immuno-blot)	Abcam	ab5417
ZO1	Invitrogen	402200,
Bestrophin1	Abcam	ab2182
MITF	Abcam	ab122982
MERTK	Abcam	ab52968
EZRIN	Santa Cruz	sc-58758
β-TUBULIN	Thermo Fisher	PA5-16863
Alexa Fluor 488 rabbit	Invitrogen	A21206
Alexa Fluor 488 mouse	Invitrogen	A21202
Alexa Fluor 647 rabbit	Invitrogen	A31573
Alexa Fluor 647 mouse	Invitrogen	A31571
HRP-rabbit secondary antibody	Invitrogen	32260
HRP- mouse secondary antibody	Thermo Fisher	62-6520
rabbit anti mouse bridging antibody	Sigma	M7023_2ML
protein A gold	CMC Utrecht	https://www.cellbiology-utrecht.nl/products.html
**Chemicals, Peptides, and Recombinant Proteins**		
soybean trypsin inhibitor	Sigma	T6522
trypsin-EDTA	Gibco	T3924
ACTIVIN A	R&D	338-AC-050
Antibiotic-Antimycotic	Gibco	15240-062
TrypLE	Thermo Fisher	12563011
protease inhibitors	Roche	4693132001
Fluorescein FITC	Invitrogen	F2182
Alexa Fluor 555	Invitrogen	A20009
Alexa Fluor 647 Phalloidin	Invitrogen	A22287
GAS6	R&D systems	885-GSB
MFGE8	R&D systems	2767-MF
FTY720	SIGMA	SML0700
Tunicamycine	SIGMA	T7765
Serum (FBS)	gibco	26140-079
trypan blue	Sigma	T8154
Paraformaldehyde	Science Services	E15714-S
Glutaraldehyde	Science Services	E16220
Hoechst 33342	Thermo Fisher	H1399
RIPA	Thermo Fisher	89900
BSA	SERVA	11926.03
10% Mini-PROTEAN® TGX™ Precast Protein Gels	BioRad	4561033
Precision Plus Protein™ WesternC™	BioRad	1610376
SuperSignal™ West	Thermo Fisher	34075
Precision Protein™ StrepTactin-HRP Conjugate	BioRad	1610380
Lowicryl K4M resin	Science Services	14330
osmium tetroxide	Science Services	19190

mTESR1	StemCell Technologies	85870
Dispase	StemCell Technologies	7923
Rock inhibitor Y-27632	StemCell Technologies	78003
**Critical Commercial Assays**		
RNeasy Mini kit	Qiagen	74106
SuperScript™ II Reverse Transcriptase	Invitrogen	18064022
SYBR™ Green PCR Master Mix	Applied Biosystems	4309155
NEBNext Poly(A) mRNA Magnetic Isolation Module	NEB	E7490L
NEBNext Ultra Directional RNA Library Prep Kit for Illumina	NEB	E7420L
XP bead purification	Beckman Coulter	A63882
VEGF Human ELISA Kit	Invitrogen	KHG0112
Qubit dsDNA HS Assay Kit	Invitrogen	Q32854
H9 human embryonic stem cell line	WiCell	WA-09
MERTK knockout in H9 (MERTK-EX2)	Previous study	DOI: 10.1016/j.stemcr.2020.02.004
MERTK knockout in H9 (MERTK-EX14)	Previous study	DOI: 10.1016/j.stemcr.2020.02.004
RP38 patient iPSC (MERTK)	Previous study	DOI: 10.1016/j.stemcr.2020.02.004
TSS isogenic iPSC control	Previous study	DOI: 10.1016/j.stemcr.2020.02.004
GraphPad Prism	La Jolla California USA,	www.graphpad.com
Knime	Berthold et al., 2009	
Cell Profiler	Lamprecht et al., 2007	
GSNAP (v2014-12-17)		http://research-pub.gene.com/gmap.
featureCounts (v1.4.6)	Liao et al., 2014	http://subread.sourceforge.net/
R		https://www.r-project.org/
DESeq2 R package (v1.6.3)	Love et al., 2014	https://bioconductor.org/packages/release/bioc/html/DESeq2.html
384 well plates	Greiner	781091
Transwells	Corning	3470
ERMN F:	SIGMA	ATGGGGATAAACCACCTGAAAAC
ERMN R:	SIGMA	GAGCAGCATGTTCCCTTGTAA
S1PR5 F:	SIGMA	GCGCACCTGTCCTGTACTC
S1PR5 R:	SIGMA	GTTGGTGAGCGTGTAGATGATG
MFSD2A F:	SIGMA	ATCAGCACCGAGCAGACTG
MFSD2A R:	SIGMA	GCTATTGAGGTCCTGGAAACAAG
Pharmakon 1600	MircoSource Discovery Systems	
Ramoplanin	SIGMA	R1781
Pyrithion Zinc	SIGMA	PHR1401
Digoxin	SIGMA	1200000
Ouabain	SIGMA	O0200000
Cephapirin Sodium	SIGMA	1102500
Potassium p-Aminobenzoate	SIGMA	A0254
Sennoside A	SIGMA	1612018
Carbidopa	SIGMA	C1335
Doxorubicin	SIGMA	1225703
Homidium Bromide	SIGMA	E8751
Merbromin	SIGMA	M7011
Succinylsulfathiazole	SIGMA	S8752

Supplemental Experimental Procedures:

Cell lines and cell culture

RPE derived from H9 hESC cell line (hESC-RPE) were used as wild-type control. Wild-type iPSC line was derived in collaboration with the stem cell and engineering facility in the CRTD. Fibroblasts were isolated from skin biopsies from RP38 patients and were reprogrammed using the non-integrating Sendai virus. RPE differentiated from patient iPSC is referred to as "MERTK-RPE" throughout the manuscript. *MERTK* gene editing with CRISPR/CAS9 was done in H9 hESC. Two modified hESC lines were obtained EX2 and EX14. Differentiated RPE are referred to as "EX2-RPE" and "EX14-RPE" throughout the manuscript. Detailed description of the generation of *MERTK* knock out cell lines, and patient iPSC line, and the characterization of the pluripotent cell lines were previously described in Almedawar et al. (Almedawar et al., 2020). All RPE cells were differentiated on transwell filters as previously described (Zhu et al., 2014, 2013) with some modifications. Briefly, dissociated stem cell colonies were counted prior to embedding in matrigel and the amount of matrigel was adjusted accordingly. Following neuroepithelial cysts trypsinization between 100,000 and 150,000 cells were plated on transwells in the presence of 5 µM rock inhibitor. Activin A concentration was reduced to 0.02 µg/ml for RPE differentiation from hESC and to 0.01 µg/ml for RPE differentiation from iPSC. RPE cells were passaged for expansion two times on transwells before use. For some experiments and as indicated in the experimental schemes, RPE cells were passaged first on transwells for expansion and then to 384-wells plates and used after 13 days. For passaging, cells on transwells were incubated with trypsin-EDTA (TE) for 10 minutes at 37ºC and 5% CO2. After incubation, cells were vigorously pipetted to obtain single cells, and transferred to a tube containing RPE medium plus soybean trypsin inhibitor. Next, RPE single cells were washed by centrifugation at 180 g for two minutes. Finally, cells were resuspended with RPE media containing activin A and 1x Antibiotic-Antimycotic, and 45,000 cells per one 384 well, or 300,000 cells per transwell were plated.

Immunofluorescence labeling of RPE cells

Cells were fixed with 4% paraformaldehyde (PFA). After several washes with PBS, they were treated with the quenching solution (1x PBS, 100 mM Glycine, 0.3% Triton X-100) for twenty minutes and then were incubated with the blocking solution (1xPBS, 1% BSA, 0.3% Triton X-100) for one hour. The primary and secondary antibodies were diluted in the blocking solution and incubated overnight at 4°C. Cell nuclei were counterstained by addition of Hoechst 33342 (1mg/ml) with the secondary antibody incubation step. The primary antibodies and their working dilutions were as follows: ZO1 (1:200), (1:500), MITF (1:500), MERTK (1:1000). The secondary antibodies Alexa Fluor 488/647 rabbit and mouse were used at 1:500 dilution. Phalloidin 647 was added to the cells after the secondary antibody (1:1000) overnight.

Isolation of porcine POS

POS were isolated from porcine eyes, as described previously (Molday et al., 1987) with some modifications. Briefly, the retinal tissue was isolated from 50 porcine eyes and homogenized in a buffer containing protease inhibitors, 0.2 mM Tris-HCl, 0.1 mM Glucose, 130 mM $NaCl_2$, 0.1 mM Taurine, and 0.02 mM $MgCl_2$. The homogenized retinal tissue was split in 6 tubes containing a sucrose gradient of 27%, 33%, 41%, 50% and 60%, and centrifuged in the Beckmann Coulter ultracentrifuge B409 at 28000 rpm for one hour at 4º C. The orange band was collected in Nalgene centrifuge tubes, and centrifuged at 13000 g for 10 minutes. Finally, the pellets were resuspended with POS storage solution containing protease inhibitors, 10 mM phosphate buffer pH 7.2, 100 mM $NaCl_2$ and 2.5% sucrose and stored at -80 ºC until they were used.

POS labeling

For labeling, Fluorescein (FITC) or Alexa Fluor 555 (AF555) were added to the POS after thawing for 1 hour at 25°C with shaking (500 rpm). Next, POS were centrifuged at 9000 g at 4°C for 10 minutes, and washed twice with the washing buffer, containing: 10% sucrose, 20 mM phosphate buffer pH7.2 and 5 mM Taurine.

Phagocytosis assay

For all experiments, cells were primed with the different treatments for 1 hour before the addition of POS. POS particles were sonicated in 500 µl RPE media containing the different treatments (MFGE8, GAS6) for 5 seconds 10% power with BRANSON Digital Sonifier 450 before addition to the cells to prevent aggregation. The composition of RPE media has been described previously(Zhu et al., 2014, 2013). Before seeding, POS were quantified using the Neubauer chamber combined with fluorescence microscopy imaging and CellProfiler analysis. In all phagocytosis experiments, unbound POS were washed away before fixation, at various time points after POS addition, depending on the purpose of the experiment, using PBS containing 1 mM $MgCl_2$ and 0.2 mM $CaCl_2$ (PBS-MC). GraphPad Prism was used for statistical significance calculations as indicated in the figure legend and final graph presentation.

Primary screening phagocytosis assay

HESC-RPE were plated in 384-wells plates for 13 days before they were used for screening. On the screening day RPE cells were primed for one hour with the library or the control treatment including MFGE8 and GAS6 (2.5 µg/ml) and DMSO only. The compounds were added to the cells in the presence of 25 µl RPE media containing 0.3125 µg/ml MFGE8 using Echo acoustic liquid handler (Beckmann Coulter). During the priming, POS were labeled with AF555 and prepared for addition. After one hour of priming 25 µl of labeled POS were added to the cells and the library compounds were added again, so that the final concentration of the compounds in the library is 10 µM. After three hours incubation at 37ºC, 5% CO2, cells were washed five times with PBS and incubated with PBS with Hoechst overnight. Plates were acquired the next day using Yokogawa's CV7000S confocal microscope. The library consisting of 1600 FDA approved compounds was distributed over nine 384-wells plates with 192 compounds each except for plate 9 which contains 64 compounds.

The screen was performed four times using four consecutive RPE preps. Besides the library, the screening plate consisted of twenty-four wells containing 0.3125 µg/ml MFGE8, DMSO (0.1%) and POS, four wells containing DMSO (0.1 %) and POS only without MFGE8, twelve wells without POS, eight wells MFGE8 and Gas6 (2.5 µg/ml) and POS, and four wells untreated (No DMSO or MFGE8) with POS.

Imaging and Image analysis

During Imaging, nine optical sections from six fields in each well were acquired, using the 63X objective, covering the different areas in the well. Acquired images were imported into the CellProfiler (Lamprecht et al., 2007) software for image analysis. POS and nuclei were segmented and values of the number, size and intensity were obtained.

Data and statistical analysis

The data obtained from image analysis was imported into KNIME (Berthold et al., 2009) for statistical analysis. The median count of the POS between the 6 images was divided by the median count of cells per well to obtain the POS/cell count. To determine positive and negative hits, log POS/cell for each well was calculated and used to calculate the z-score using the following formula:

$$z - score = \frac{Log\frac{POS}{cell}\ treatment - Mean\ Log\frac{POS}{cell}DMSO}{SD\ DMSO}$$

Compounds that gave a z-score of ≥+3 were considered as positive hits and compounds that gave a z-score of ≤-3 were considered negative hits.

To calculate the coefficient of variation (CV), the following formula was used:

$$CV = \frac{Mad\ POS/cell}{Median\ POS/cell}X100$$

Hits confirmation:

For confirming the obtained hits the hits were reordered as powder from Sigma-Adrich and added to the cells with and without POS with three different concentrations (5 µM, 10 µM, 15 µM) in 384-wells plates (confirmation I) and then in transwell plates (confirmation II). Confirmed hits were subjected to further validation using secondary assays.

Orthogonal assays

Western Blot based phagocytosis assay:

To distinguish total and internalized POS by means of western blot, half of the samples were treated with 2 mM EDTA for 10 minutes to remove bound POS from the cells before lysis and the other half was left with PBS as described previously (Mao and Finnemann, 2013). Around one million cells were lysed with RIPA buffer for 30 minutes at 4°C. Next, the protein lysates were separated from the pellet by centrifugation and around 50 µg of protein was loaded into Mini-PROTEAN® TGX™ Precast Protein Gels. 10 µl of Precision Plus Protein™ WesternC™ was loaded as a standard. Proteins were transferred into PVDF membrane using TE70 Semi-dry transfer unit. Blocking was done for one hour at room temperature in 5% milk dissolved in TBST. To analyze POS phagocytosis by means of western blot, membranes were incubated with mouse anti-RHO antibody (1:1000), which recognizes the C-terminal of the protein, followed by anti-rabbit β-TUBULIN (1:1000) antibody overnight at 4 °C in 5% milk-TBST. HRP-mouse secondary antibody (1:1000) and HRP-rabbit secondary antibody (1:1000) were added for one hour at room temperature in 5% milk-TBST. Precision Protein™ StrepTactin-HRP Conjugate was also added with the secondary to view the standard in chemiluminescence mode. The blot membrane was incubated with SuperSignal™ West according to Manufacturer´s instructions and imaged with LAS4000.

Fluorescence based phagocytosis assay in transwells

RPE cells grown in transwells were cut out from the well, transferred on slides and imaged using SP5-MP confocal microscope. During imaging thirty-one optical sections in three fields per well were acquired.

Trypan blue based phagocytosis assay

To distinguish bound and internalized POS by means of fluorescence imaging, 0.4% trypan blue or PBS were added to RPE cells in 384-wells plates for 10 minutes and then washed six times with PBS to remove leftover dye before methanol fixation at -20 ºC, as described previously (Almedawar et al., 2020; Mao and Finnemann, 2013). Trypan blue quenches bound POS in treated wells and allows the detection of internalized POS only. CV7000S confocal microscope was used for imaging.

FACS based phagocytosis assay:

To monitor the effect of RM on POS degradation AF555-labeled POS were added to the cells. After three hours the cells were washed and RM (20 µM) or Chloroquine (50 µM) were added to the cells for twenty-one more hours. Cells were then trypsinized and subjected to FACS sorting using Amnis® ImageStream®^{X} Mk II Imaging Flow Cytometer. Trypsinization detached bound POS allowing visualization of internalized POS as described in (Westenskow et al., 2012).

Scanning Electron Microscopy (SEM)

RPE cells on transwell filters were fixed with modified Karnovsky's solution, containing 2% glutaraldehyde (GA) plus 2% PFA in 0.1 M phosphate buffer pH 7.4, until processing. For processing, samples were washed with PBS and post-fixed in 1% osmium tetroxide in PBS for two hours on ice. Next, they were washed with water and dehydrated in a graded series of ethanol starting from 30% and up to 100%. Then, they were critical-point dried using the Leica CPD 300 (Leica Microsystems, Vienna, Austria), cut out from the transwell, mounted on 12 mm aluminium stubs, and sputter-coated with gold using the Baltec SCD 050 (Leica Microsystems, Vienna, Austria). Finally, filters were analyzed with a Jeol JSM

7500F cold field emission SEM (Jeol, Eching, Germany) at 5 kV acceleration voltage using the lower secondary electron detector.

Transmission Electron Microscopy (TEM)

For immune-EM of POS seeded on RPE, the samples were fixed in 4% PFA. After several washes in PBS, the filters with the cells were dissected with a razor blade into small pieces (1-2 mm) and dehydrated and infiltrated in Lowicryl K4M resin using the progressive lowering of temperature (PLT) method (Carlemalm et al., 1982). After polymerisation, the blocks were raised up to room temperature, and 70 nm sections were cut on a Leica UC6 ultramicrotome. Sections were mounted onto copper mesh grids for immunolabeling. On-section labeling of ultrathin sections was performed as previously described (Fabig et al., 2012). In brief, sections were blocked with 1% BSA in PBS, incubated for one hour with primary antibodies mouse anti-RHO (1:200) in BSA/PBS. Next, they were washed with PBS, and incubated with bridging antibody (rabbit anti mouse, 1:100 in BSA/PBS) for thirty minutes. Sections were then washed with PBS, incubated with protein A gold (1:100) for one hour in BSA/PBS, washed with PBS, post-fixed in 1% glutaraldehyde/PBS, washed with water, stained with 4% uranyl acetate in water, washed with water and dried for TEM inspection. Ultrastructure and gold labeling was imaged with a FEI Morgagni 268D or a Jeol JEM1400 Plus both at 80 kV acceleration voltage.

Next-generation sequencing (RNA-seq)

HESC-RPE cultured in transwells were pretreated with either 20 μM RM or 0.2% DMSO in the presence of 0.3125 μg/ml MFGE8 for 1 hour. Then, POS were seeded on the cells and incubated for 3 hours before they were lysed and total RNA was purified from three consecutive RPE differentiation rounds, using Qiagen RNeasy Mini kit. Control samples included the same treatment without POS and untreated samples. Using the NEBNext Poly(A) mRNA Magnetic Isolation Module, polyadenylated mRNA was enriched from 1μg total RNA with an integrity number of ≥ 9, according to the manufacturer's instructions. The mRNA was eluted in 15 μl 2x first strand cDNA synthesis buffer (NEBNext), in order to chemically fragment the samples, followed by reverse transcription, second strand synthesis, end repair, A tailing and adapter ligation according to the manual of NEBNext Ultra Directional RNA Library Prep Kit for Illumina. For ligation, hybridized custom adaptors were used (Adaptor-Oligo 1 and 2). Afterwards, excess of non-ligated adapters were depleted by an XP bead purification, adding 1x bead. Finally, samples were indexed during a PCR enrichment step with 15 cycles of amplification using primers carrying a specific index sequence indicated with 'NNNNNN' (Index Primer1, 2 and 3). After two more XP beads purifications (0.9x) libraries were quantified using Fragment Analyzer Standard Kit. For Illumina flowcell production, samples were equimolarly pooled and then sequenced on two Illumina NextSeq flowcells in 75bp single-end mode.

Oligonucleotide sequence	SOURCE
Adaptor-Oligo 1: 5'-ACA CTC TTT CCC TAC ACG ACG CTC TTC CGA TCT 3'	This Study
Adaptor-Oligo 2: 5'-P-GAT CGG AAG AGC ACA CGT CTG AAC TCC AGT CAC-3'	This Study
Index Primer1: AAT GAT ACG GCG ACC ACC GAG ATC TAC ACT CTT TCC CTA CAC GAC GCT CTT CCG ATC T	This Study
Index Primer2: GTG ACT GGA GTT CAG ACG TGT GCT CTT CCG ATC T	This Study
Index Primer3: CAA GCA GAA GAC GGC ATA CGA GAT NNNNNN GTG ACT GGA GTT)	This Study

Resulting reads were mapped with GSNAP (v2017-08-15) to the human genome (hg38) using splice junction information from Ensembl (v81) as support. Uniquely mapped reads were then converted into counts per gene using featureCounts (v1.5.3) and gene annotations from Ensembl (v81). Normalization of the raw counts based on the library size and testing for differential expression between the different cell types/treatments was performed with the DESeq2 R package (1.18.1). Genes with adjusted p-value (Benjamini-Hochberg) less than 0.05 were considered differentially expressed. The heat maps presented in Figure 6 were generated using GraphPad Prism. Images in Figure S7 and data in Table S7 were generated using Ingenuity Pathway Analysis software (IPA).

Quantitative PCR (QPCR)

HESC-RPE cultured in transwells were pretreated with either 20 μM RM or 0.2% DMSO in the presence of 0.3125 μg/ml MFGE8 for 1 hour. Then, POS were seeded on the cells and incubated for 3 hours before they were lysed and total RNA was purified from three consecutive RPE differentiation rounds, using Qiagen RNeasy Mini kit. For reverse transcription, 500 ng of RNA were incubated with random primers and dNTP Mix for 5 minutes at 65 ºC. Next, SuperscriptII RT enzyme, 5X first strand buffer, DTT and RNAse out were added to the reaction. Primer hybridization, reverse transcription and enzyme deactivation were performed in the thermocycler according to the following program: 25 ºC for 10 minutes, 42 ºC for 50 minutes, 70 ºC for 15 minutes. For the qPCR 1.5 μl of the cDNA was added to the reaction containing SYBR™ Green PCR master mix S1PR5, POLR2A, MSFD2A and ERMN primers according to manufacturer's instructions. Quantitative PCR was performed on three biological experimental repeats and nine technical repeats, which include three cDNAs and from each cDNA three wells, per condition.

Transepithelial resistance (TER) measurement

TER was measured before treatment with RM and controls, and 3, 6, and 24 hours following treatment with the EVOM^2 using STX2 electrodes. Represented TER values were calculated as follows:

$$TER\ (\Omega * cm^2) = \left(Sample\ TER\ (\Omega) - Blank\ TER\ (\Omega)\right) \times\ Area\ cm^2$$

Whereby, blank is a transwell coated with GFR matrigel, and contains the same amount of media as the sample, and the area of the used transwells is 0.03 cm^2.

VEGF ELISA based Measurement

Media from the upper chamber and the lower chamber of the transwells where RPE cells were cultured was collected and frozen at -80 ºC before treatment and twenty-four hours after treatment with RM and controls. Media was diluted 1:5, and VEGF ELISA was performed according to manufacturer's instructions.

References

Almedawar, S., Vafia, K., Schreiter, S., Neumann, K., Khattak, S., Kurth, T., Ader, M., Karl, M.O., Tsang, S.H., Tanaka, E.M., 2020. MERTK-Dependent Ensheathment of Photoreceptor Outer Segments by Human Pluripotent Stem Cell-Derived Retinal Pigment Epithelium. Stem Cell Reports 14, 374–389.

Berthold, M.R., Cebron, N., Dill, F., Gabriel, T.R., Kötter, T., Meinl, T., Ohl, P., Thiel, K., Wiswedel, B., 2009. KNIME - the Konstanz information miner. ACM SIGKDD Explor. Newsl. 11, 26.

Carlemalm, E., Garavito, R.M., Villiger, W., 1982. Resin development for electron microscopy and an analysis of embedding at low temperature. J. Microsc. 126, 123–143.

Fabig, G., Kretschmar, S., Weiche, S., Eberle, D., Ader, M., Kurth, T., 2012. Labeling of Ultrathin Resin Sections for Correlative Light and Electron Microscopy. Methods Cell Biol. 111, 75–93.

Lamprecht, M.R., Sabatini, D.M., Carpenter, A.E., 2007. CellProfiler: Free, versatile software for automated biological image analysis. Biotechniques 42, 71–75.

Mao, Y., Finnemann, S.C., 2013. Analysis of Photoreceptor Outer Segment Phagocytosis by RPE Cells in Culture. Methods Mol. Biol. 935, 285–295.

Molday, R., Hicks, D., Molday, L., 1987. Peripherin . A Rim-Specific Membrane Protein of Rod Outer Segment Discs. Invest. Ophthalmol. Vis. Sci. 28, 50–61.

Westenskow, P.D., Moreno, S.K., Krohne, T.U., Kurihara, T., Zhu, S., 2012. Using Flow Cytometry to Compare the Dynamics of Photoreceptor Outer Segment Phagocytosis in iPS-Derived. Invest. Ophthalmol. Vis. Sci. 53, 6282–6290.

Zhu, Y., Carido, M., Meinhardt, A., Kurth, T., Karl, M.O., Ader, M., Tanaka, E.M., 2013. Three-Dimensional Neuroepithelial Culture from Human Embryonic Stem Cells and Its Use for Quantitative Conversion to Retinal Pigment Epithelium. PLoS One 8, e54552.

Zhu, Y., Schreiter, S., Tanaka, E.M., 2014. Accelerated Three-Dimensional Neuroepithelium Formation from Human Embryonic Stem Cells and Its Use for Quantitative Differentiation to Human Retinal Pigment Epithelium. Methods Mol. Biol. 1307, 345–355.
