# Supplementary material for: A Human Retinal Pigment Epithelium-Based Screening Platform Reveals Inducers of Photoreceptor Outer Segments Phagocytosis
Source: Stem Cell Reports. 2020 Nov 25;15(6):1347–61. doi: 10.1016/j.stemcr.2020.10.013 (PMC7724476; doi:10.1016/j.stemcr.2020.10.013)
Supplement: Document S2. Article plus Supplemental Information [file mmc6.pdf]

Stem Cell Reports
Article
ISSCR
OPEN ACCESS

A Human Retinal Pigment Epithelium-Based Screening Platform Reveals Inducers of Photoreceptor Outer Segments Phagocytosis

Sven Schreiter,[1,6] Katerina Vafia,[1,6] Rico Barsacchi,[2] Stephen H. Tsang,[4] Marc Bickle,[2] Marius Ader,[1] Mike O. Karl,[1,3] Elly M. Tanaka,[5] and Seba Almedawar[1,6,*]

[1]Technische Universität Dresden, Center for Molecular and Cellular Bioengineering (CMCB), Center for Regenerative Therapies Dresden (CRTD), Fetscherstraße 105, 01307 Dresden, Germany
[2]Max Planck Institute of Molecular Cell Biology and Genetics, Pfotenhauerstraße 108, 01307 Dresden, Germany
[3]German Center for Neurodegenerative Diseases (DZNE) Dresden, Tatzberg 41, 01307 Dresden, Germany
[4]CUMC/Edward S. Harkness Eye Institute, 635 West 165th Street, New York, NY 10032, USA
[5]Research Institute of Molecular Pathology (IMP), Vienna Biocenter (VBC), Campus Vienna Biocenter 1, 1030 Vienna, Austria
[6]These authors contributed equally
*Correspondence: seba.almedawar@tu-dresden.de or siba84@gmail.com (S.A.)
https://doi.org/10.1016/j.stemcr.2020.10.013

SUMMARY

Phagocytosis is a key function in various cells throughout the body. A deficiency in photoreceptor outer segment (POS) phagocytosis by the retinal pigment epithelium (RPE) causes vision loss in inherited retinal diseases and possibly age-related macular degeneration. To date, there are no effective therapies available aiming at recovering the lost phagocytosis function. Here, we developed a high-throughput screening assay based on RPE derived from human embryonic stem cells (hRPE) to reveal enhancers of POS phagocytosis. One of the hits, ramoplanin (RM), reproducibly enhanced POS phagocytosis and ensheathment in hRPE, and enhanced the expression of proteins known to regulate membrane dynamics and ensheathment in other cell systems. Additionally, RM rescued POS internalization defect in Mer receptor tyrosine kinase (MERTK) mutant hRPE, derived from retinitis pigmentosa patient induced pluripotent stem cells. Our platform, including a primary phenotypic screening phagocytosis assay together with orthogonal assays, establishes a basis for RPE-based therapy discovery aiming at a broad patient spectrum.

INTRODUCTION

Millions of people around the world suffer from vision loss due to retinal degeneration to which no efficient therapeutic treatment exists. The function of the retina relies on robust photoreceptor outer segment (POS) phagocytosis by the underlying retinal pigment epithelium (RPE). Thus, any dysfunction in POS phagocytosis leads to vision loss (Strauss, 2005). Indeed, dysfunctional phagocytosis has been described in both acquired age-related macular degeneration (AMD) (Golestaneh et al., 2017; Inana et al., 2018) and genetically inherited retinal degenerative diseases, including retinitis pigmentosa (RP) caused by mutations in Mer receptor tyrosine kinase (MERTK) (Almedawar et al., 2020; Niemann et al., 2000) and bestrophinopathies (Zorych et al., 2017). Thus, enhancing POS phagocytosis in the RPE might be of therapeutic relevance to many retinal degenerative diseases, which can be applied as a standalone therapy or in combination with gene- and cell-based therapies to enhance their outcomes.

The outer membrane tips of the photoreceptors accumulate photo-oxidized material, which needs to be recycled by the RPE on a daily basis to prevent accumulation of waste in the interphotoreceptor space (Strauss, 2005). During the initial steps of phagocytosis, POS expose phosphatidylserines (PSs) on their outer membranes, similar to apoptotic cells (Finnemann and Silverstein, 2001). RPE apical membrane, containing actin-rich ensheathing membranes, recognizes the so-called "eat-me" signals presented on the membrane of POS through bridging opsonins such as milk fat globule-EGF8 (MFGE8) and growth arrest-specific protein 6 (GAS6), which bind to their respective RPE membrane receptors αVβ5 integrin (Nandrot et al., 2007) and MERTK (Hall et al., 2001; Law et al., 2015), initiating POS fragmentation and internalization (Almedawar et al., 2020). Following internalization, POS undergo lysosomal degradation followed by recycling of some of the components such as fatty acids stemming from docosahexaenoic acid (DHA) and retinoic acid back to the photoreceptors (Strauss, 2005). Mutations in the *MERTK* gene lead to RP disease marked by early-onset vision loss. The lack of efficient therapy for RP highlights the need for new strategies to test potential therapeutic targets and/or compounds.

Target-based screens have been classically used by the pharma industry to identify lead compounds and study their modulatory effect on presumptive targets. These assays rely mostly on cell-free assays, or on reporter cells that allow the testing of hundreds of thousands of compounds, in a hypothesis-driven approach that can overlook the complexities of cellular responses. Phenotypic drug screens are instead target agnostic and focus more on the exploration of phenotypic space in the context of relevant cellular disease models, by identifying relevant phenotypes, which then require target deconvolution campaigns

This is an open access article under the CC BY license (http://creativecommons.org/licenses/by/4.0/).

(Swinney, 2013). Phenotypic screens have the potential to determine toxicity of compounds before moving into further downstream assays (Priest and Erdemli, 2014). To our knowledge, such a high-throughput phenotypic screen for enhancers of POS phagocytosis has not yet been performed using human embryo-derived RPE cells that can be produced in unlimited amounts and bear resemblance in their function and phenotype to primary human RPE.

Up to 2013, over 12,000 new drug applications have been approved by the US Food and Drug Administration (FDA), leading to around 1,500 new molecular entities (Kinch et al., 2014). The Pharmakon 1600 from MircoSource Discovery Systems is a selection of 1,600 compounds that reached clinical evaluation and are well characterized. Screening of FDA-approved compounds has the potential to identify small molecules that can be repurposed to treat inherited and acquired retinal degenerative diseases.

Here we combine phenotypic screening technologies based on a highly relevant human cellular disease model in the form of RPE derived from healthy and diseased human pluripotent stem cells (hPSCs) to propose a robust workflow that goes from the identification to the validation of small molecules that improve phagocytosis function in healthy RPE cells and rescue phagocytosis deficiency in MERTK mutant RPE, which reproduce a pathology that causes vision loss.

RESULTS

Human RPE in a Miniaturized 384-Well Plate Assay Are Functional and Express Mature RPE Markers

We developed a miniaturized POS phagocytosis assay suitable for high-throughput screening, based on RPE cells derived from human embryonic stem cells (hESCs) cultured in 384-well plates, and we checked their quality and relevant functionality. We observed that RPE cells express mature RPE markers in a polarized fashion (Figure 1A) similar to what we previously observed in the more physiological transwell culture plate format (Almedawar et al., 2020). We further sonicated the POS, as previously described (Almedawar et al., 2020), to obtain a more homogeneous POS size, which facilitates image analysis (Figures 1B and 1C). Then, we titrated the POS by seeding them on RPE cells for 3 h based on our findings in Almedawar et al. (2020). Our aim was to choose a concentration that is higher than the lowest concentration, and yet does not saturate the cells, to be able to capture any increase in POS phagocytosis during the compound's screening. To this end, we determined a concentration of 10^6 POS/mL (Figure 1D). Next, we defined the kinetics of POS uptake in the transwell system by western blot and we saw that the peak of POS uptake occurs at 3 h following POS addition (Figures 2A and 2B), which is in line with what we previously observed (Almedawar et al., 2020). In the 384-well plate, RPE cells phagocytose POS in a receptor-ligand-dependent manner at 3 h (Figure 2C). In the presence of FBS, which is a natural source for GAS6 and MFGE8 phagocytosis ligands, RPE cells take up more POS with time (Figure 2D) and are able to partially degrade them (Figures 2E and 2F). These results show that RPE cells are able to phagocytose and degrade POS in the 384-well format similar to what has been observed in the transwell format (Almedawar et al., 2020). Finally, to determine the optimal number of RPE cells in the screening assay, we plated the cells in different densities and challenged them with the same amount of POS. We observed that cells seeded with a density of 45,000 cells/well showed the highest number of phagocytosed POS at 3 h (Figures 2G and 2H). These experiments allowed us to establish the experimental conditions of our primary screening assay, which consists of hESC-derived RPE seeded at 45,000 cells/well density for 13 days, and challenged with sonicated POS at a concentration of 10^6/mL for 3 h. MFGE8 and GAS6 are two phagocytosis ligands that are usually externally provided to RPE cells to induce specific POS phagocytosis (Almedawar et al., 2020; Law et al., 2015). A combination of MFGE8 and GAS6 at 2.5 μg/mL concentration was used as positive control and DMSO as vehicle control. In order to focus the hits on compounds that enhance phagocytosis independently of MERTK activation, GAS6 (MERTK ligand) was not supplemented in the test wells that received the library. Instead, low concentrations of MFGE8 (0.3125 μg/mL), which activates αVβ5 integrin phagocytosis receptor, were supplemented to maintain ligand-mediated specific POS phagocytosis. Prior to addition of POS, cells were pretreated or primed for 1 h with MFGE8 and GAS6 in the control wells (2.5 μg/mL each) and MFGE8 (0.3125 μg/mL) together with the FDA library compounds in the test wells.

FDA-Approved Compounds Screen Reveals Stimulators of Phagocytosis in a Human RPE Assay

The MicroSource Discovery Systems Pharmakon library, containing 1,600 FDA-approved compounds, was selected for the primary screening assay, which was performed in quadruplicates. The hits were repurchased and confirmed in three concentrations in the 384-well and transwell format. Next, orthogonal assays in wild-type and MERTK mutant RPE cells were performed to validate the hits. Finally, to exclude toxic effects of the hits in the RPE, physiological assays were performed *in vitro*. In Figure 3A the screening platform pipeline is demonstrated. Using automated image analysis for identification and counting of POS and cells (Figure 3B), followed by automated statistical

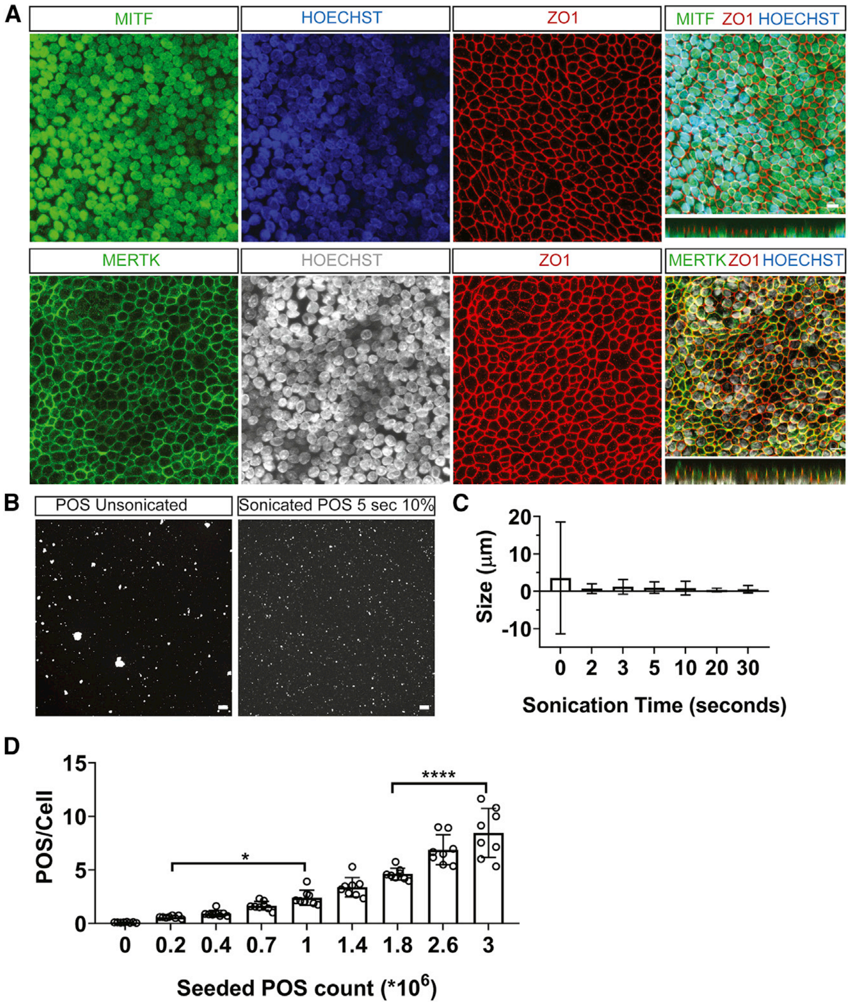

Figure 1. Human RPE Cells Cultured in Miniaturized Format Express Mature RPE Markers Are Polarized and Functional

(A) Immunofluorescence images of hESC-RPE that are plated in 384-well plates at a density of 45,000 cells/well for 13 days. Cells express mature RPE markers including MITF and MERTK. They also show polarized expression pattern of ZO1 and MERTK as shown in the orthogonal views.

(B) POS were subjected to different sonication times with 10% intensity to obtain homogeneous size distribution before addition to the cells. Before sonication, big and small POS clumps were observed in Alexa Fluor 555 (AF555)-labeled POS. After sonication for 5 s, POS clumps appeared more uniform.

(C) Quantification of fluorescence images of AF555-labeled POS after sonication. Data are represented as the mean of the size of POS particles ± SD. Sonication for 5 s with 10% intensity was chosen to be used in the phagocytosis assay.

(D) Increasing concentrations of POS were added to hESC-RPE in 384-well plates. Data represent the mean of the total POS/cell count ±SD. N = 4 RPE differentiation batches (two wells per batch). Significance was calculated using one-way ANOVA comparing all samples with each other. Addition of 10^6 POS/well increases the number of total POS/cell significantly compared with the first concentration of 2×10^5.

analysis of *Z* scores, we could identify and confirm hits. Compounds with a *Z* score lower than or equal to −3 were chosen as potential hits that decrease POS count, reflecting either a decrease in POS phagocytosis or an increase in POS degradation rate (Figure 3C). In contrast, compounds with a *Z* score higher or equal to +3 were chosen as hits that increase POS count, reflecting increased phagocytosis (Figure 3D). After the first confirmation testing in a 384-well plate, we eliminated several compounds, which were auto-fluorescent (doxorubicin, homidium bromide, merbromin). Other eliminated compounds, including carbidopa and cephapirin sodium, did not maintain a *Z* score of more than or equal to +3. In contrast, potassium p-aminobenzoate and sennoside A did not maintain a negative *Z* score of less than or equal to −3, and thus were excluded from further validation. We observed a decrease in the nuclei number in cells treated with succinylsulfathiazole, which suggested that it might be toxic to RPE cells and was discarded from follow-up assays. Thus, from the first confirmation round, we could confirm two positive hits, ramoplanin (RM) and pyrithione zinc (PZ), and two negative hits, digoxin and ouabain. We observed a dose response for both RM and PZ. However, PZ showed an increase in POS phagocytosis compared with DMSO only at 20 μM and not at 10 μM, which was the concentration that we used in the screen (Figure 3E). In the primary screen, our positive control significantly increased POS phagocytosis compared with baseline control conditions including DMSO alone, DMSO with 0.3125 μg/mL MFGE8, and untreated (Figure S1A). When looking at the distribution of individual wells, we observed that the *Z* scores of the screening library wells were widely distributed over the *Z* score axis, while those of DMSO were limited between −3 and +3 and showed a flatter distribution closer to 0. In contrast, wells that were treated with MFGE8 and GAS6 showed a *Z* score between 0 and equal to or larger than +3 (Figure S1B). Within the DMSO wells, we identified 0.001% (2 out of

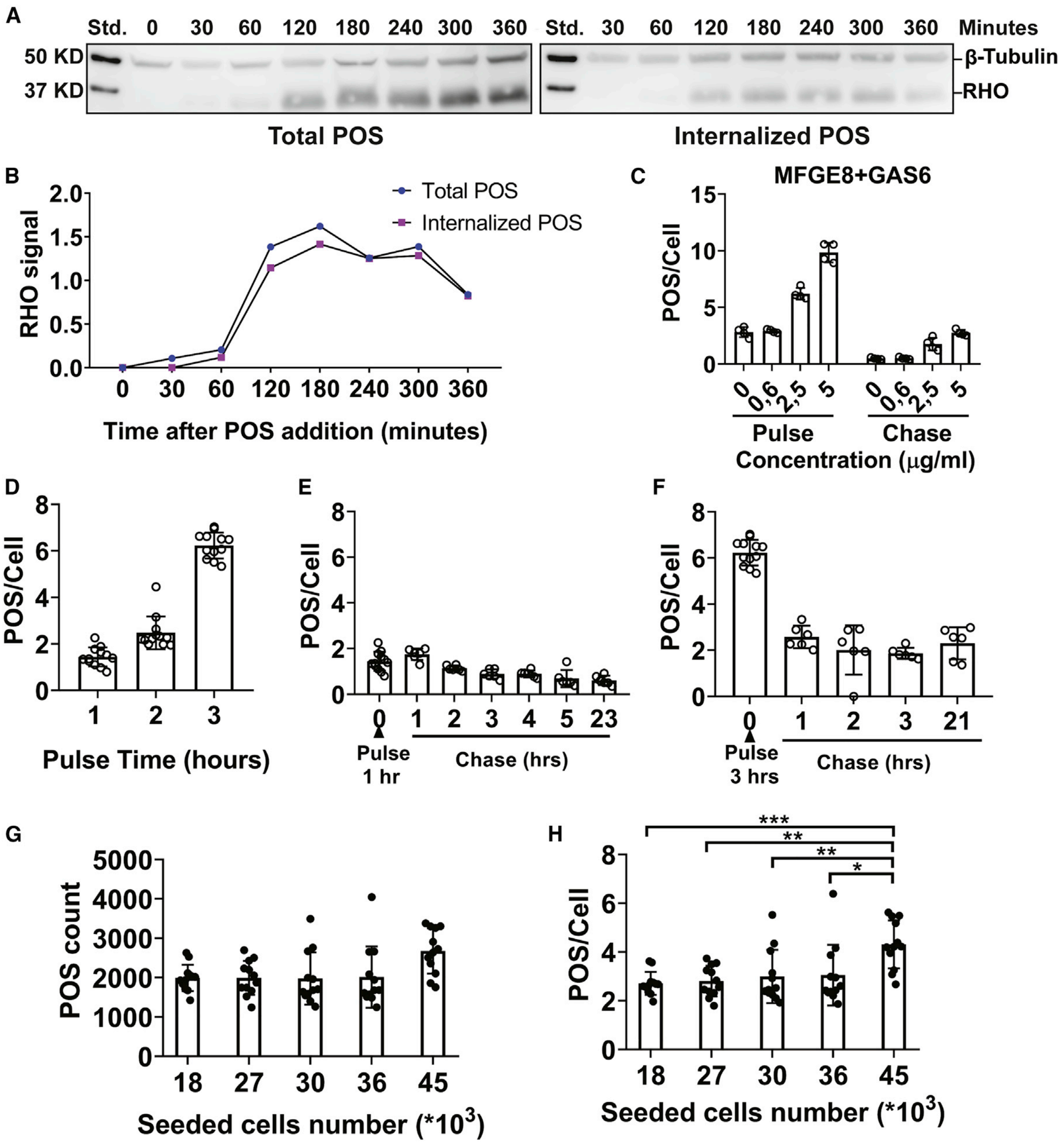

Figure 2. Kinetics of Miniaturized POS Phagocytosis Assay Uptake and Degradation by Human RPE

(A) Western blot analysis of total and internalized POS by hESC-RPE over the course of 6 h. POS were added to RPE for 0, 30, 60, 120, 180, 240, 300, and 360 min and then cells were washed and treated with EDTA to detach bound POS and monitor internalized POS or with PBS to monitor total POS. Finally, cells were lysed and analyzed by western blot. Membranes were blotted with anti- RHO to monitor POS and β-tubulin as a loading control.

(B) Analysis of the western blot images in (A). Maximum binding and internalization is reached at 3 h following POS addition.

(C) POS were added to hESC-RPE in 384-well plates for 3 h in the presence of increasing concentrations of phagocytosis ligands, MFGE8+GAS6. At 3 h cells were either washed and fixed or left for 6 h more to monitor degradation of POS. Data represent the mean of the total POS/cell count ±SD. N = 4 RPE differentiation batches. RPE cells respond to ligand stimulation in a dose-dependent manner and they are able to degrade POS after 6 h.

(D) AF555-labeled POS were added to hESC-RPE for 1, 2, and 3 h and then cells were washed and fixed to monitor increase in total phagocytosed POS over time. Data represent the mean of the total POS/cell count ±SD. N = 4 RPE differentiation batches (three wells per batch).

(legend continued on next page)

1,093 DMSO wells) false-positive and 0.0009% (1 out of 1,093 DMSO wells) false-negative wells (Figure S1C). The assay showed an intraplate variation of around 10% and an interplate variation of around 20% (Figure S1D).

RM Increases POS Phagocytosis by Human RPE

The second confirmation step was carried out in RPE cells cultured in transwells, where we also confirmed the positive effect of RM and PZ on POS phagocytosis in a dose-dependent manner (Figure S1E). Further downstream assays showed that induction of POS phagocytosis by RM was stable across several RPE differentiation rounds (Figure S1F). Addition of RM to both RPE and POS showed an additive effect compared with individual treatments (Figure S2A). We also detected that increasing the amount of seeded POS increases the amount of phagocytosed POS in both RM and DMSO conditions (Figure S2B). However, the largest signal (RM) to noise (DMSO) ratio was observed in wells seeded with 10^6 POS, which was the concentration used during the screen (Figure S2C). In the absence of phagocytosis ligands, RM increased POS phagocytosis in a dose-dependent manner up to 25 μM in two wild-type pluripotent stem cell lines. With 5 μM a significant increase could be already observed compared with DMSO (Figure S2D). In the presence of phagocytosis ligands, a significant increase was only observed when 10 μM RM was added (Figure S2E).

The increase in POS/cell count in RPE cells when treated with RM might be a result of blocking POS internalization or degradation. To exclude this possibility, we monitored POS internalization and degradation in the presence of RM, DMSO, or chloroquine, which is known to block lysosomal degradation by elevating lysosomal pH (Klionsky et al., 2016), using three different methods: fluorescence-based imaging (Figure 4A), fluorescence-activated cell sorting (FACS) (Figure 4B), and western blot (Figures 4C–4F). We observed using all methods that RM does not block internalization, or degradation of internalized POS.

Tight monolayer formation and polarized vascular endothelial growth factor (VEGF) secretion are two essential physiological aspects of the RPE that regulate the blood-retina barrier. Healthy RPE cells secrete VEGF mainly to the basal side and thus exhibit a ratio of the basally secreted to the apically secreted VEGF that is greater than one. To measure the effect of RM on RPE health, we designed a set of experiments (Figure S3A) where we could verify the positive effect of RM on POS phagocytosis by fluorescence microscopy (Figure S3B) and measure VEGF secretion (Figures S3C and S3D) and transepithelial resistance (TER) (Figures S3E and S3F). We confirmed that RPE cells maintained a high basal/apical VEGF secretion ratio (Figures S3C and S3D) and a high TER value (Figures S3E and S3F) after 24 h of treatment with RM and POS. These assays validated the positive effect of RM on POS phagocytosis in wild-type RPE cells and excluded side effects on RPE healthy physiological functions.

RM Upregulates Apical Membranes Processes Extension Regulators and Stimulates Ensheathment

We performed RNA sequencing (RNA-seq) analysis to determine which genes are upregulated following treatment with RM and POS for 3 h (Figures 5A and 5B). The three top genes with the highest differential gene expression score compared with DMSO were the actin-stabilizing protein (ERMN), the transporter for the essential ω-3 fatty acid DHA (MFSD2A), and the S1P receptor 5 (S1PR5), which is involved in photoreceptors progenitors' proliferation and apical processes formation, and is regulated by DHA. Upregulation of these three genes was also confirmed by means of quantitative PCR (Figure 5C). We observed that inhibition of S1PR5 rather than MFSD2A by small molecules inhibited RM's effect on POS phagocytosis (Figure 5D). These results suggest that RM might be enhancing POS phagocytosis by stabilizing actin through ERMN upregulation and by upregulating S1PR5, which possibly stimulates apical RPE processes formation and POS ensheathment. Notably, apical processes formation is key in POS ensheathment, and participates in POS phagocytosis by fragmenting POS particles prior to internalization and is affected in retinal degenerative diseases (Almedawar et al., 2020). Thus, we examined the effect of RM on POS ensheathment in the presence of the phagocytosis ligands using SEM in healthy RPE cells. Strikingly, RM upregulates POS ensheathment and leads to elongated microvilli (red arrows in Figure 6). Thus, the RNA-seq analysis revealed interesting gene targets that might be connected

(E and F) In the same experiment as in (D), separate wells were washed at 1 or 3 h and kept for 1, 2, 3, 4, 5, and 23 h or 1, 2, 3, and 21 h respectively to monitor POS degradation overtime.

(G) hESC-RPE cells were seeded on 384-well plates in increasing concentrations to obtain the optimal cell number for the phagocytosis assay. POS (10^6 POS/well) were seeded on the cells for 3 h before they were fixed and imaged. Data represent the mean of the total POS count ±SD. N = 3 RPE differentiation batches (three wells per batch). (H) Total POS count shown in (G) was divided over the nuclei count to obtain POS/cell count. Data represent the mean of the total POS/cell count ±SD. Significance was calculated using one-way-ANOVA comparing all samples with each other. Plating 45,000 cells/well increases the number of POS/cell significantly compared with the other concentrations.

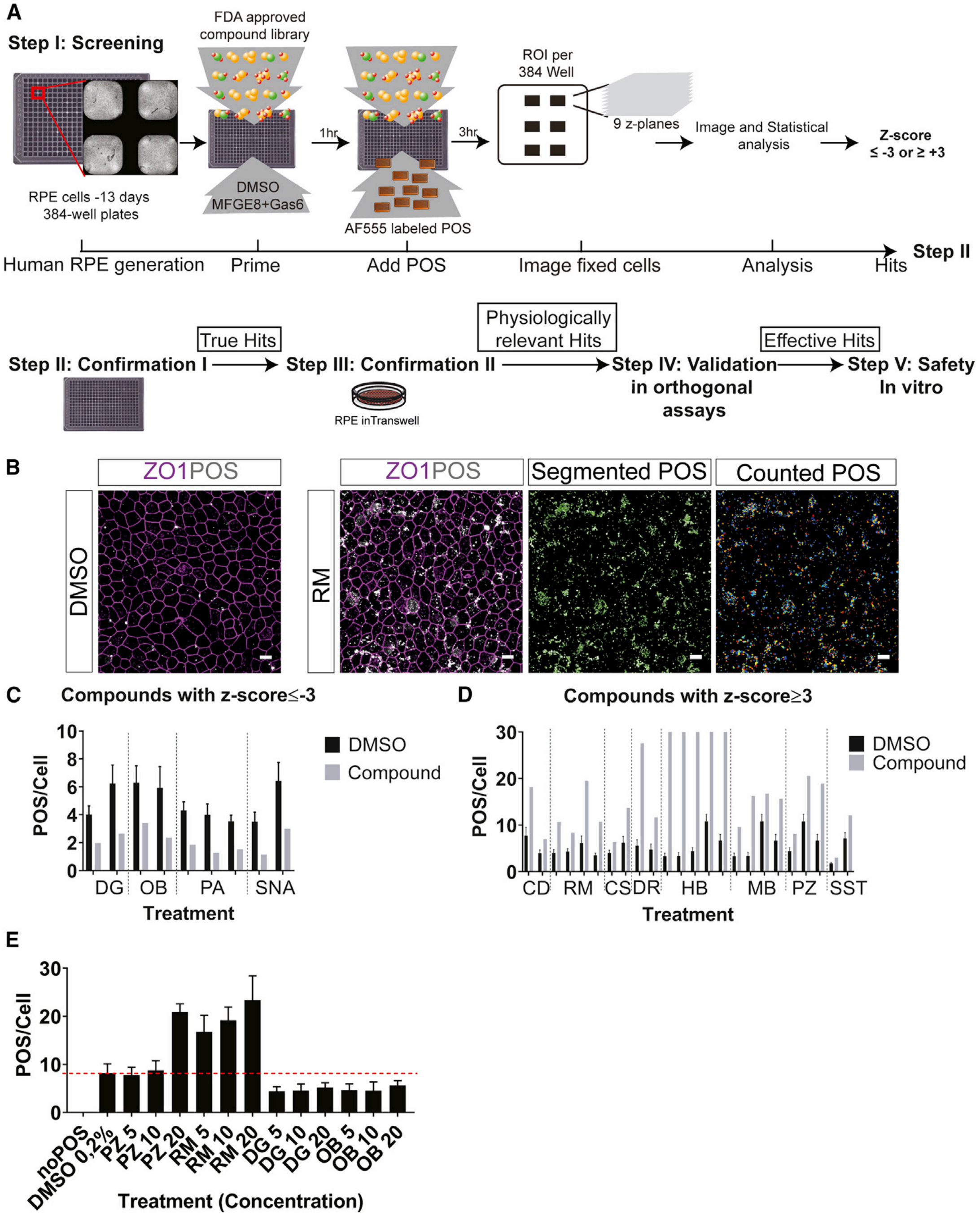

Figure 3. Ramoplanin Increases POS Phagocytosis by Human RPE *In Vitro*

(A) Screening and hits confirmation pipeline. Refer to Table S1 for library details.

(B) Fluorescence images of hESC-RPE challenged with AF555-labeled POS (gray) and RM/DMSO for 3 h. Fixed cells were labeled with ZO1 (magenta) to mark the boarders of the cells. Images were uploaded to the cell profiler software, which gives a count of the segmented POS particles. Images of RM-treated cells show an increase in the number of POS particles (bound and internalized) compared with DMSO (vehicle). Scale bar: 10 μm.

(C and D) Hits were determined based on the obtained *Z* score. In (C) POS/cell count for the hits that obtained a *Z* score of ≤ -3 in at least two of the four runs are shown in comparison with the DMSO control. In (D) POS/cell count for the hits that obtained a *Z* score of $\geq +3$ at least in two of the four runs are shown in comparison with the DMSO control. Related to Table S2. CD, carbidopa; CS, cephapirin sodium; DG, digoxin; DR, doxorubicin; HB, homidium bromide; MB, merbromin; PZ, pyrithione zinc; OB, ouabain; PA, potassium p-aminobenzoate; RM,

(legend continued on next page)

to key processes regulating RPE phagocytosis, and that could be considered for therapy development for diseases where phagocytosis is defective. Here, we provide strong evidence that RM stimulates the POS ensheathment that is defective alongside phagocytosis in RPE cells of RP patients suffering from vision loss due to a mutation in the MERTK gene.

Ramoplanin Rescues Internalization Defect in Human MERTK Mutant RPE In Vitro

To specifically address whether RM rescues the internalization defect seen in MERTK mutant RPE *in vitro*, we examined POS phagocytosis by means of western blot analysis on RPE cells derived from three MERTK mutant hPSCs described earlier by our laboratory (Almedawar et al., 2020). The MERTK mutant induced pluripotent stem cells (iPSCs) were derived from an RP patient with homozygous MERTK deletion spanning exons 6–19, and leading to complete loss of expression of MERTK (MERTK-RPE). Expression of MERTK was rescued by inserting MERTK under the CMV promoter in the MERTK locus (isogenic control). We also generated by means of CRISPR/CAS9 technology a homozygous knockout in the MERTK gene spanning exons 2–19, which also leads to complete loss of MERTK expression (EX2-RPE) and in exons 14–19 (EX14-RPE), which leads to partial loss of expression. We observed that RM rescues the internalization defect observed in all MERTK mutant RPE (Figures 7A and 7B). To determine the EC50 (effective concentration giving half-maximal response) of RM in wild-type and MERTK mutant RPE, by means of fluorescence-based imaging we added increasing doses of RM to RPE in the presence of POS. RM increased POS phagocytosis in a dose-dependent manner across wild-type and MERTK mutant RPE up to 20 ± 5 μM (Figures 7C and 7D). Using trypan blue to bleach externally bound POS, we observed that RM increases the number of internalized POS in a dose-dependent manner in wild-type and EX2-RPE (MERTK mutant RPE) (Figures 7E and 7F). These results were confirmed by means of confocal fluorescence imaging in the transwell format, where we could observe more POS in the higher z-planes on the apical surface in DMSO-treated MERTK mutant RPE cells (Figures S4A–S4E). In contrast, in RM-treated MERTK mutant RPE, most of the POS were detected in lower z-planes of the RPE, indicating higher internalization. By means of transmission electron microscopy, we detected internalized POS which are rhodopsin (RHO) positive in EX2-RPE treated with RM but not with DMSO (Figures S4F and S4G). Thus, ramoplanin stimulates phagocytosis in healthy hRPE and rescues the internalization defect seen in MERTK mutant RPE in a dose-dependent manner.

DISCUSSION

In this work we describe the establishment of a phenotypic screening platform based on a cell model that is highly relevant to retinal degeneration pathology, namely RPE. The primary screen was followed by orthogonal assays and an RPE cell model that recapitulates RP disease. Using this platform, we identified and validated RM as a top hit that increases POS phagocytosis in wild-type RPE and rescues phagocytosis defect in MERTK-deficient RPE cells. Using additional orthogonal assays, we determined that RM does not block POS internalization or degradation and does not have negative effects on the tight monolayer formation or polarized secretion of VEGF in RPE cells *in vitro*.

Phenotypic screens usually provide contextual information of the relevant cell type, but exhibit higher variation than target-based assays. In our screening assay, we detected up to 10% intraplate variation within different wells of a 384-well plate, and up to 20% interplate variation of plates done on different days, which falls within the variation range expected for a phenotypic screen (Iversen et al., 2012). We assume that RPE differentiation per se did not account for the observed variation, as RPE derived during various differentiation rounds showed stable phagocytosis induction in the presence of RM, and had similar transcriptional profiles. We aimed at reducing the source of variation during POS production by pooling different POS isolations, which reduced differences in POS quality and count between different days. To minimize further differences during POS labeling, we used one reconstituted Alexa Fluor 555 (AF555) vial throughout the screen. However, there were some factors that we could not control, such as changes in the laser intensity due to replacement or microscope maintenance. For this reason, we defined the hits in our screen using the *Z* score, which is a widely known statistical measurement and is a useful tool to standardize data over a range of experiments (Malo et al., 2006). It is based on a SD distance over the mean of the control (DSMO), and it is calculated for each individual compound in the

ramoplanin; SNA, sennoside A; SST, succinylsulfathiazole. Data are represented as the mean ± SD. n = 24 wells DMSO per plate (36 plates in total) and N = 4 plates from four consecutive RPE differentiation batches with one well per plate for each compound. (E) Confirmation in 384-well plates (step II). Only confirmed hits are shown in three different concentrations (5, 10, and 20 μM). Data are represented as the mean ± SD. N = 2 RPE differentiation batches with six wells/batch for each compound and concentration. Related to Figures S1 and S2, Table S3 (compound autofluorescence in the 555 channel), S4 (*Z* score of the compounds in the first confirmation), and S5 (list of hits from confirmation II in transwells).

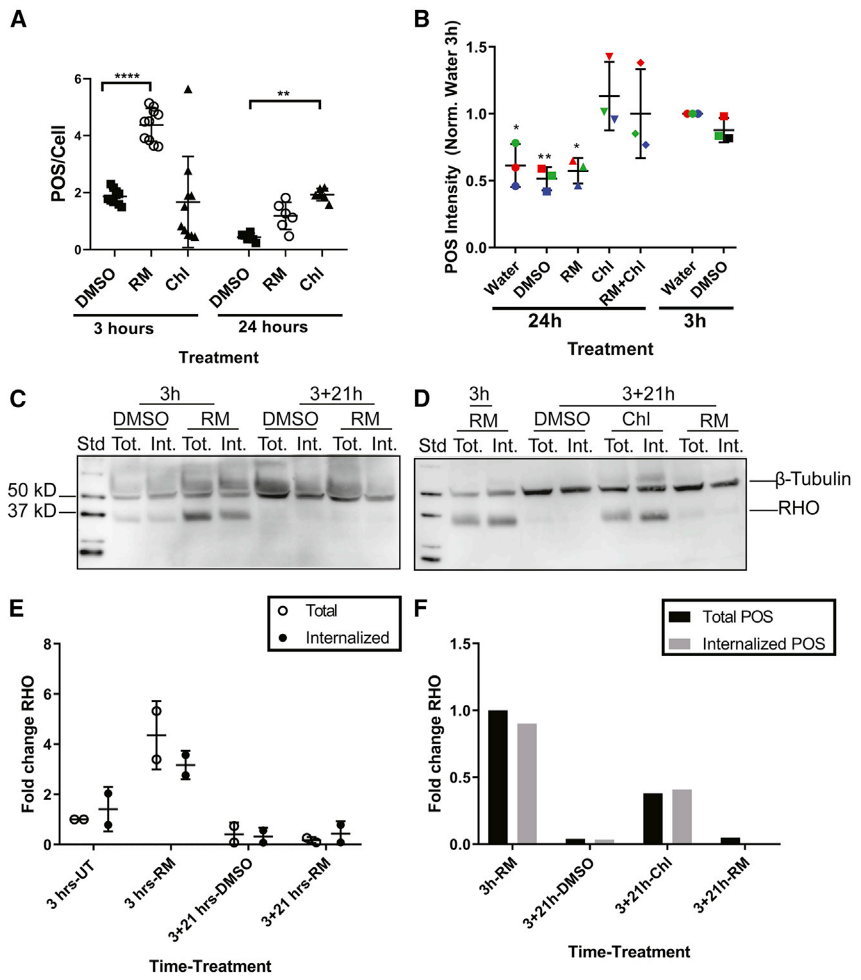

Figure 4. Ramoplanin Does Not Block POS Internalization or Degradation by Human RPE

(A) AF555-labeled POS were added to hESC-RPE for 3 h in the presence of 10 μM RM or 50 μM chloroquine (Chl). Alternatively, POS were added for 3 h without treatment and then unbound POS were washed off and RM and Chl were added for 21 h to monitor POS degradation. Cells were fixed and analyzed with confocal fluorescence microscopy. RM increases total POS at 3 h and does not block degradation after 24 h. In contrast, Chl blocks POS degradation after 24 h. Data are represented as the mean ± SD. N = 3 RPE differentiation batches (two wells per batch). Significance was calculated using two-way ANOVA. Samples within the same time point were compared with DMSO. ns, $p > 0.05$; *$p < 0.05$; **$p < 0.01$; ***$p < 0.001$; ****$p < 0.0001$.

(B) AF555-labeled POS were added to hESC-RPE for 3 h in the presence of 10 μM RM or 50 μM Chl. Alternatively, POS were added for 3 h without treatment and then unbound POS were washed off and RM and Chl were added for 21 h to monitor POS degradation. Cells were incubated with trypsin-EDTA to dissociate them and detach bound POS. The intensity of internalized POS in single cells was analyzed with FACS. RM does not block POS degradation after 24 h. In contrast, Chl blocks POS degradation after 24 h. Addition of RM to Chl does not have an additive effect. Intensities were normalized to water at 3 h within each experiment. Different colors represent different biological replicates (N = 3 repeats performed on different days). Data are represented as the mean ± SD. Significance was calculated using two-way ANOVA. Samples were compared with water at 3 h ns, $p > 0.05$; *$p < 0.05$; **$p < 0.01$; ***$p < 0.001$; ****$p < 0.0001$.

(C) POS were added to hESC-RPE for 3 h in the presence of 10 μM RM or 0.01% DMSO. Alternatively, POS were added for 3 h without treatment and then unbound POS were washed off and RM and DMSO were added for 21 h to monitor POS degradation. Cells were incubated either with PBS to monitor total POS (Tot.), or with EDTA to dissociate bound POS and monitor internalized POS (Int.). Cell lysates were analyzed with western blot. Membranes were probed with RHO and β-tubulin. RM does not block POS degradation after 24 h, while Chl-treated cells show defects in POS degradation.

(D) POS were added to RPE cells for 3 h in the presence of 10 μM RM. Alternatively, POS were added for 3 h without treatment and then unbound POS were washed off and RM, DMSO, or Chl (50μM) were added for 21 h to monitor POS degradation. Cell lysates were analyzed with western blot similar to (C). In contrast to Chl, RM does not block POS degradation after 24 h.

(E) Analysis of (C). Values are normalized to total POS 3 h untreated (UT). Data are represented as the mean ± SD. N = 2 RPE differentiation batches (two wells per batch).

(F) Analysis of (D). Values are normalized to total POS 3 h RM. In Figure S3, further data on the effect of RM on RPE and retina health are shown *in vitro*.

same plate. We used a cutoff of ±3; i.e., a 3-fold DMSO SD distance of the compound over the mean of the DMSO control and a p value of 0.003. Following hit confirmation, we obtained two positive hits, which is similar to what has been reported in the literature for phenotypic screens. For instance, Naska et al. (2016) screened the Prestwick library and LOPAC-Sigma library, which together have 2,400 compounds, and obtained three confirmed hits.

The second confirmed best hit identified in our screen, PZ, is a coordination complex of zinc and shows antibacterial and antifungal activity. It has been used to treat dandruff and seborrheic dermatitis. It inhibits fungal

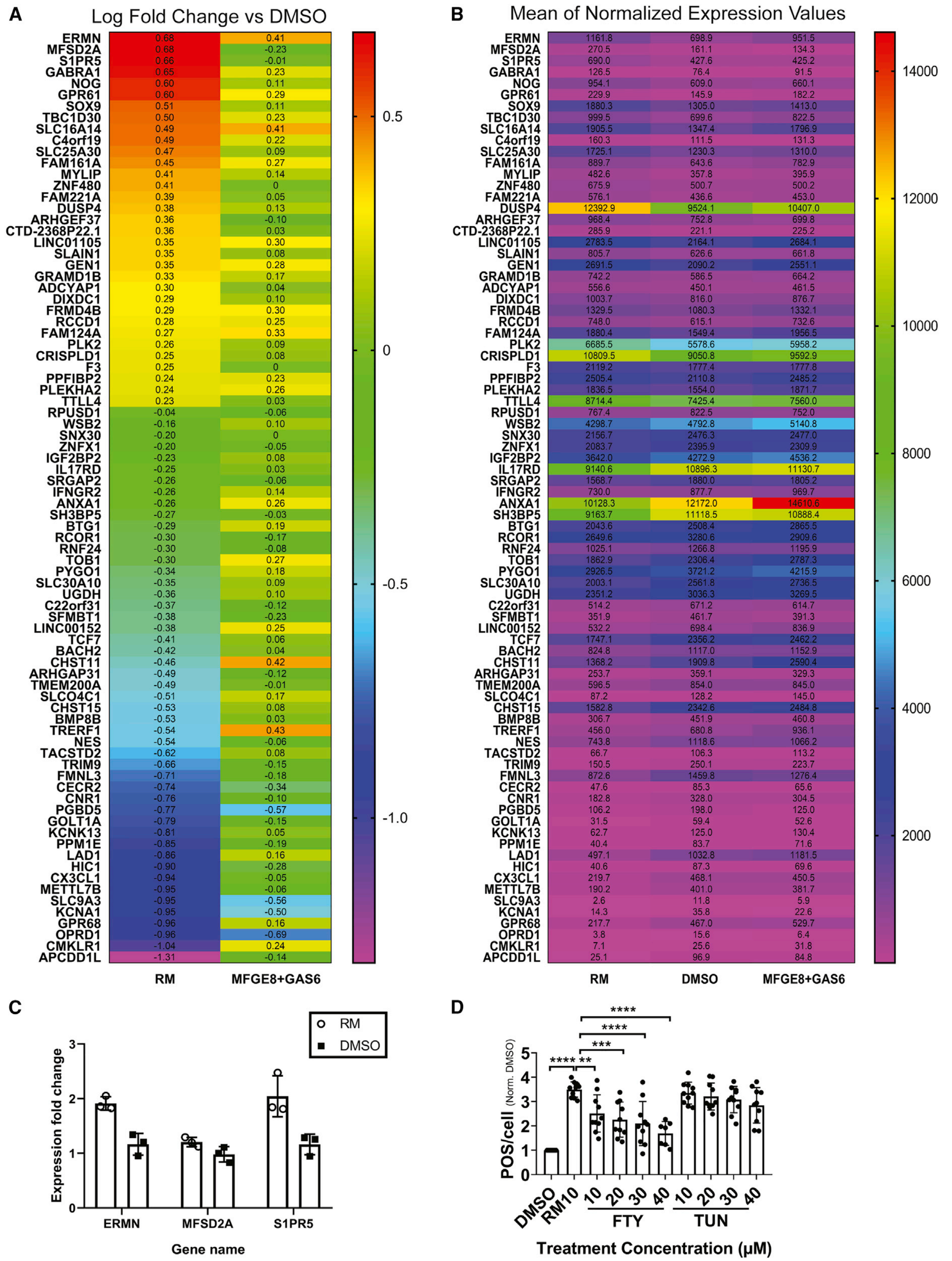

(legend on next page)

growth by increasing cellular levels of copper, which damages iron- sulfur clusters of proteins essential for fungal metabolism (Reeder et al., 2011; Schwartz, 2016). The use of PZ is not limited to its antimicrobial effect. It has been shown that it activates voltage-gated potassium channels (Kv7) and hyperpolarizes the membrane of rat pulmonary artery smooth muscle cells resulting in airway relaxation (Eid and Gurney, 2018). It also decreased apoptosis, caspase-3 activation, and infarct size following ischemia/reperfusion (Thokala et al., 2017). The beneficial effect of PZ has been attributed to replenishing zinc levels, which are decreased following ischemia. Interestingly, oral supplementation of zinc to macular degeneration patients has shown beneficial therapeutic effect (Vishwanathan et al., 2013). Thus, the positive activity of this compound might be due to the presence of zinc in its center. Alternatively, it might activate phagocytosis by activating potassium channels on the membrane of RPE cells, resulting in its hyperpolarization. Indeed, activation of potassium channels might be involved in POS phagocytosis through regulation of the cell volume (Müller et al., 2014; Wimmers et al., 2007).

We focused our downstream validation efforts on the more potent hit RM, a glycolipodepsipeptide antibiotic, which interferes with the glycosylation of peptidoglycans in bacteria, inhibiting cell wall synthesis and making it active against multidrug-resistant gram-positive aerobic and anaerobic bacteria (Fang et al., 2006). The upregulated genes uncovered by RNA-seq analysis of cells treated with RM revealed interesting future drug targets. The top three upregulated genes in RM-treated RPE cells, which were additionally confirmed by qPCR, were ERMN, MFSD2A, and S1PR5. Expression of these genes was upregulated in RM-treated RPE even in the absence of POS, as observed by RNA-seq analysis, which suggests that their upregulation is due to RM treatment only. ERMN binds to F-actin and is known to enhance its polymerization, participating in cell motility or processes elongation. ERMN was first identified in oligodendrocytes, where it localizes to the outer cytoplasmic lip of the myelin sheath and participates in the late stages of mature nerves myelination (Brockschnieder et al., 2006). ERMN expression has been also detected in olfactory ensheathing cells and is involved in enlargement of processes that ensheath individual axons in the fila olfactoria (Tang et al., 2009). The process of POS ensheathment by RPE cells bears analogy to the above-described processes and has been shown to participate in POS fragmentation and internalization (Almedawar et al., 2020). In this work, we also observed by SEM that RM enhances POS ensheathment. This suggests that ERMN might participate in POS ensheathment in RPE cells and its upregulation might lead to enhanced phagocytosis. ERMN expression has been detected in primary rat RPE, but not in cultured ARPE-19 cells (Liang et al., 2018). To our knowledge, this is the first report of ERMN expression in *in vitro* cultured RPE cells, which supports the notion that pluripotent stem cell-derived RPE cells are a better-suited model for phagocytosis assays than commercially available cell lines. F-actin morphology has been shown to be predictive of phagocytic capacity of RPE cells (Müller et al., 2018). Following RM treatment, no stress fibers were detected, as indicated by phalloidin staining, suggesting that RM has a positive effect on actin morphology and dynamics, which are involved in POS phagocytosis.

The second upregulated gene, MFSD2A, is a transporter of DHA (docosahexaenoic acid), which is highly enriched in the photoreceptors' membrane discs. MFSD2A is expressed by the RPE and is involved in transporting DHA from the recycled POS and from the blood stream to the photoreceptors (Wong et al., 2016). Upregulation of MFSD2A by RM in RPE cells suggests upregulation not only of the phagocytosis process but also of the recycling of essential fatty acids back to the photoreceptors participating in their regeneration. Blocking MFSD2A by a small molecule inhibitor (Wang et al., 2016) did not significantly reduce the effect of RM on phagocytosis, which

Figure 5. Ramoplanin Upregulates Expression of ACTIN Networks Stabilizing Protein ERMN and Surface Proteins in Human RPE

(A) A heatmap of log2 of fold change in gene expression in RM (20 μM) and POS, or MFGE8+GAS6 (2.5 μg/mL) and POS, compared with DMSO (0.2%) and POS treated samples. Upregulated and downregulated genes with p value less than 0.01 are shown. N = 3 biological repeats. Related to Tables S6 and S7. ERMN MFSD2A and S1PR5 are upregulated in the presence of RM compared with DMSO. ERMN also seems to be upregulated in MFGE8+GAS6 treatment. MFSD2A and S1PR5 upregulation seems to be specific for RM treatment.

(B) A heatmap of the mean of normalized expression values of the same genes in (A) in RM and POS, or MFGE8+GAS6 and POS, or DMSO and POS treated samples. Related to Table S6. RNA-seq data were deposited in GEO (https://www.ncbi.nlm.nih.gov/geo/). Reference number: GSE132828.

(C) Upregulation of ERMN, S1PR5, and MFSD2A expression in RPE cells treated with RM and POS was confirmed using quantitative PCR analysis. Data are represented as the mean ± SD. n = 27 technical repeats (three RPE wells, three cDNA, three wells from each cDNA). N = 3 biological repeats performed on different days.

(D) Inhibition of S1PR5 diminishes RM's positive effect on phagocytosis. FTY720 is a known inhibitor of S1PR5 at high concentrations. Tunicamycine (TUN) is a known inhibitor of MFSD2A. Data are represented as the mean ± SD. N = 2 RPE differentiation batches (five wells per batch).

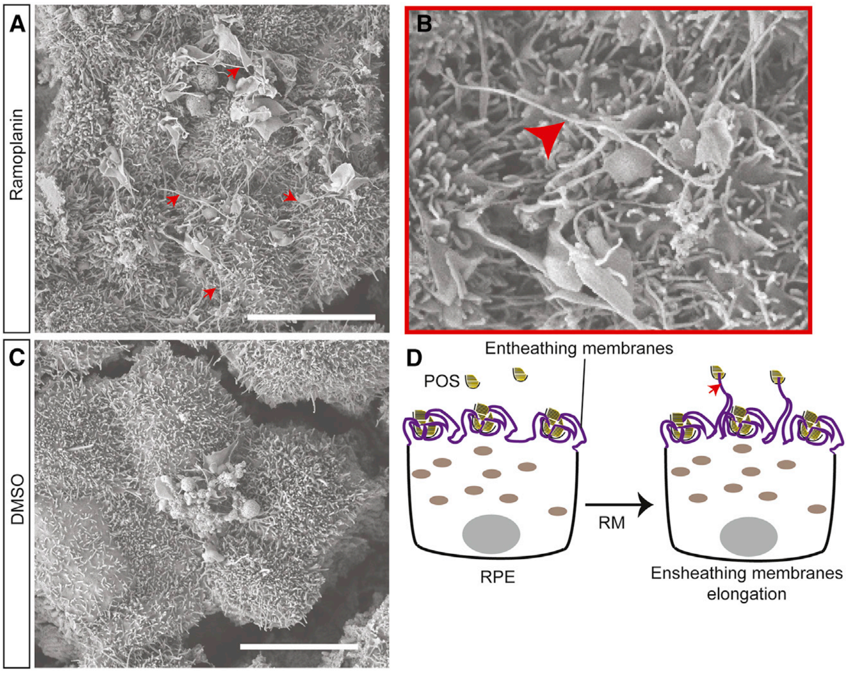

Figure 6. Ramoplanin Enhances POS Ensheathment

(A–C) SEM images of hESC-RPE treated with DMSO/RM and POS in the presence of FBS. RM enhances POS ensheathment by healthy human RPE cells. Red arrows point at elongated microvilli. Scale bar: 10 μm.

(D) A scheme of observed effects of RM on POS ensheathment.

suggests that MFSD2A might not be the primary target of RM.

S1PR5 is one of the five sphingosine 1-phosphate receptors (S1PRs) known for sphingosine-1-phophate (S1P) (Vestri et al., 2017), which is involved in photoreceptors' initial proliferation and differentiation (Miranda et al., 2009). S1P also stimulates cytoskeletal and membrane remodeling, including the formation of apical photoreceptor processes, and enhances opsin and peripherin expression and promotes their localization to these processes. Interestingly, synthesis of S1P is indirectly upregulated by DHA through sphingosine kinase upregulation (Miranda et al., 2009). FTY720 is a known agonist of S1PR5 at nanomolar concentrations. However, at higher concentrations it seems to downregulates expression of S1P receptors (Fryer et al., 2012; Valentine et al., 2010). In this work, inhibition of S1PR5 by FTY720 leads to decreased POS phagocytosis in the presence of RM, eliminating RM's positive effect on phagocytosis, which suggests that RM might exert its effects through S1PR5 upregulation. To our knowledge, the RNA-seq dataset described in this work is the first dataset that describes expression profiling of RPE during POS phagocytosis in default and upregulated states, and can be used to identify more interesting gene targets involved in this process. Additionally, it reveals insights into potential regulators of POS ensheathment that are still incompletely understood.

In conclusion, we have established an RPE-based screening platform, through which we have identified interesting compounds and targets, which can facilitate the identification of further therapeutic compounds for retinal degenerative diseases. This platform differs from other previously established ones (Cai et al., 2019) in that it is entirely based on hPSC-derived RPE, which are of higher quality and can be equally scalable, compared with commonly used RPE cell lines, and can reproduce human disease phenotype (Almedawar et al., 2020). The setup of the primary screening and orthogonal assays can be easily scaled up and adapted to pharmaceutical industry standards to be screened using novel and larger compounds libraries. Our data indicate that RM derivatives and/or target process, particularly POS ensheathment, might represent untapped therapeutic compounds and targets respectively for RP patients and others.

EXPERIMENTAL PROCEDURES

Cell Lines and Cell Culture

RPE derived from H9 hESC cell line (hESC-RPE) were used as wild-type control. The wild-type iPSC line was derived in collaboration with the stem cell and engineering facility in the Center for Regenerative Therapies, Dresden (CRTD). Generation and characterization of MERTK mutant RPE cells were previously described in Almedawar et al. (2020). All RPE cells were differentiated on transwell filters as previously described (Zhu et al., 2013), with some modifications. Briefly, dissociated stem cell colonies were counted prior to embedding in matrigel and the amount of matrigel was adjusted accordingly. Following neuroepithelial cysts trypsinization, between 100,000 and 150,000 cells were plated on transwells in the presence of 5 μM rock inhibitor. Activin A

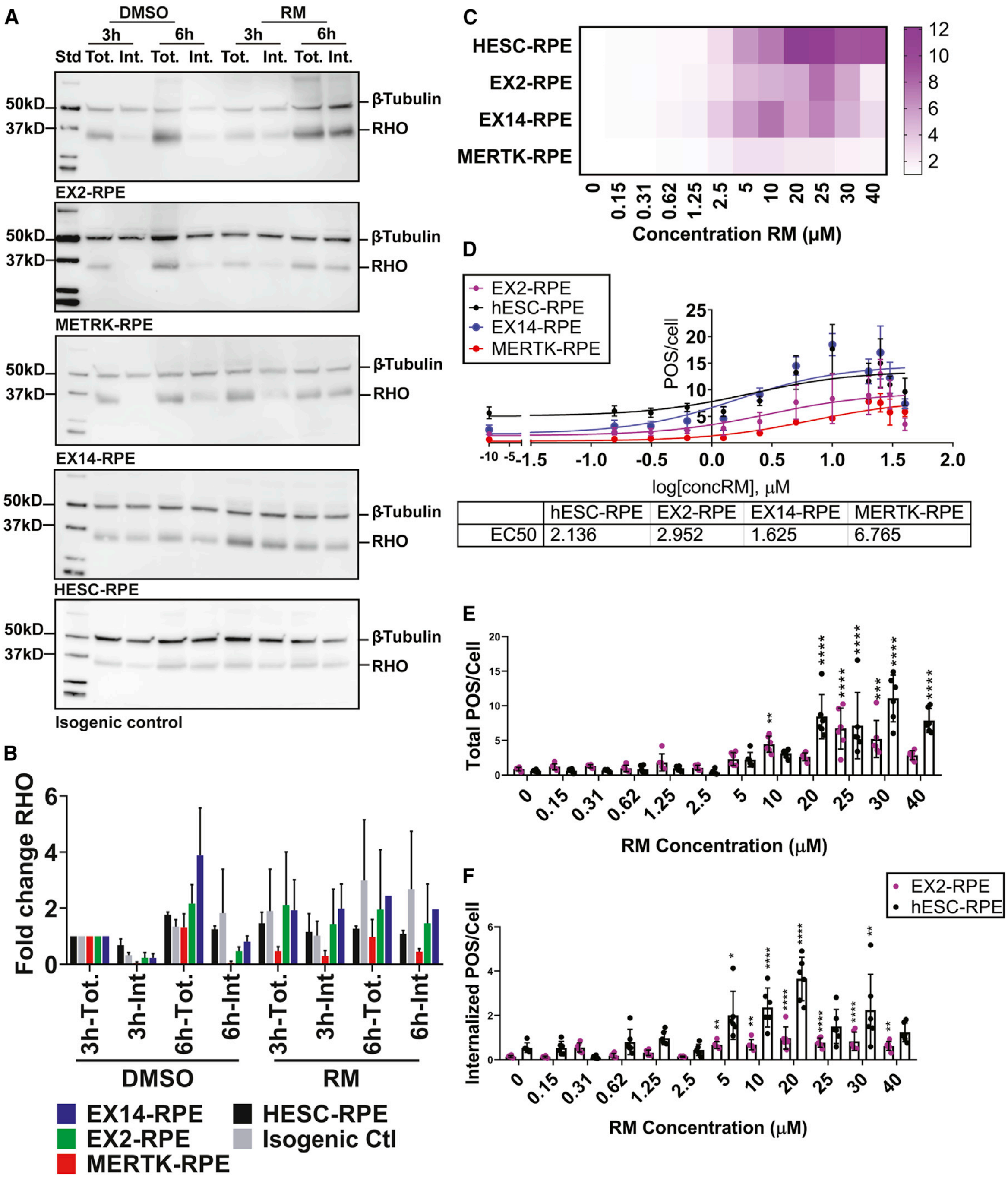

Figure 7. Ramoplanin Rescues Internalization Defect of Human MERTK Mutant RPE in a Dose-Dependent Manner

(A) RM rescues internalization defect in human MERTK mutant RPE determined by means of western blot of total and internalized POS by hESC-RPE, and *MERTK* mutant RPE at 3 and 6 h after addition of POS and RM/DMSO. Cells were washed and treated with EDTA to detach bound POS and monitor internalized (Int.) POS or with PBS to monitor total (Tot.) POS. Finally, cells were lysed and analyzed by western blot. Membranes were blotted with anti- RHO to monitor POS and β-tubulin as a loading control.

(B) Analysis of (A) shows that MERTK mutant RPE have internalization defect, which is rescued by the addition of RM. N = 3 RPE differentiation batches. Repeats were performed on different days. Related to Figure S4.

(C) Increasing concentrations of RM were added, in the presence of AF555-labeled POS for 3 h, to hESC-RPE and RPE cells differentiated from *MERTK* mutant iPSC (MERTK-RPE) and hESC (EX2-RPE and EX14-RPE). At 3 h, cells were washed and analyzed with confocal fluorescence microscopy. Higher concentrations of RM increase the total POS/cell count in all RPE lines. Data are represented as

(legend continued on next page)

concentration was reduced to 0.02 μg/mL for RPE differentiation from hESC and to 0.01 μg/mL for RPE differentiation from iPSC. RPE cells were passaged for expansion two times on transwells before use. For some experiments, and as indicated in the experimental schemes, RPE cells were passaged first on transwells for expansion and then to 384-well plates and used after 13 days. For passaging, cells on transwells were incubated with trypsin-EDTA (TE) for 10 min at 37°C and 5% CO_2. After incubation, cells were vigorously pipetted to obtain single cells, and transferred to a tube containing RPE medium plus soybean trypsin inhibitor. Next, RPE single cells were washed by centrifugation at 180 *g* for 2 min. Finally, cells were resuspended with RPE media containing Activin A and 1× antibiotic-antimycotic, and 45,000 cells per 384-well plate, or 300,000 cells per transwell were plated.

Isolation of Porcine POS and Labeling

POS were isolated from porcine eyes based on our protocol described in Almedawar et al., 2020. For labeling, fluorescein isothiocyanate (FITC) or AF555 were added to the POS after thawing for 1 h at 25°C with shaking (500 rpm). Next, POS were centrifuged at 9,000 *g* at 4°C for 10 min, and washed twice with the washing buffer containing 10% sucrose, 20 mM phosphate buffer at pH7.2, and 5 mM taurine.

Phagocytosis Assay

For all experiments, cells were primed with the different treatments for 1 h before the addition of POS. POS particles were sonicated in 500 μL of RPE media containing the different treatments (MFGE8, GAS6) for 5 s at 10% power with BRANSON Digital Sonifier 450 before addition to the cells to prevent aggregation. The composition of RPE media has been described previously (Zhu et al., 2013). Before seeding, POS were quantified using the Neubauer chamber combined with fluorescence microscopy imaging and CellProfiler analysis. In all phagocytosis experiments, unbound POS were washed away before fixation, at various time points after POS addition, depending on the purpose of the experiment, using PBS containing 1 mM $MgCl_2$ and 0.2 mM $CaCl_2$ (PBS-MC). GraphPad Prism was used for statistical significance calculations as indicated in the figure legend and final graph presentation.

Primary Screening Phagocytosis Assay

HESC-RPE were plated in 384-well plates for 13 days before they were used for screening. On the screening day, RPE cells were primed for 1 h with the library or the control treatment including MFGE8 and GAS6 (2.5 μg/mL) and DMSO only. The compounds were added to the cells in the presence of 25 μL of RPE media containing 0.3125 μg/mL MFGE8 using Echo acoustic liquid handler (Beckmann Coulter). During the priming, POS were labeled with AF555 and prepared for addition. After 1 h of priming, 25 μL of labeled POS were added to the cells and the library compounds were added again, so that the final concentration of the compounds in the library was 10 μM. After 3 h incubation at 37°C, 5% CO_2, cells were washed five times with PBS and incubated with PBS with Hoechst overnight. Plates were acquired the next day using Yokogawa's CV7000S confocal microscope. The library consisting of 1,600 FDA-approved compounds was distributed over nine 384-well plates with 192 compounds each, except for plate 9, which contained 64 compounds. The screen was performed four times using four consecutive RPE preps. Besides the library, the screening plate consisted of 24 wells containing 0.3125 μg/mL MFGE8, DMSO (0.1%), and POS; four wells containing DMSO (0.1%) and POS only without MFGE8; 12 wells without POS; eight wells with MFGE8 and Gas6 (2.5 μg/mL) and POS; and four wells untreated (no DMSO or MFGE8) with POS. The outer wells were not included in the assay to exclude edge effect.

Imaging and Image Analysis

During imaging, nine optical sections from six fields in each well were acquired, using the 63× objective, covering the different areas in the well. Acquired images were imported into the CellProfiler (Lamprecht et al., 2007) software for image analysis. POS and nuclei were segmented and values of the number, size, and intensity were obtained.

Data and Statistical Analysis

The data obtained from image analysis was imported into KNIME (Berthold et al., 2009) for statistical analysis. The median count of the POS between the six images was divided by the median count of cells per well to obtain the POS/cell count. To determine positive and negative hits, log POS/cell for each well was calculated and used to calculate the *Z* score using the following formula:

$$z-score=\frac{Log\,\frac{POS}{cell}\,treatment - Mean\,Log\,\frac{POS}{cell}\,DMSO}{SD\,DMSO}$$

Compounds that gave a *Z* score of ≥+3 were considered as positive hits and compounds that gave a *Z* score of ≤ −3 were considered negative hits.

To calculate the coefficient of variation (CV), the following formula was used:

$$CV = \frac{Mad\,POS/cell}{Median\,POS/cell}X100$$

the mean ± SD. N = 3 RPE differentiation rounds for MERTK-RPE (one well per batch) and N = 3 RPE differentiation rounds for the other RPE lines (two wells per batch).

(D) Dose-response fitted curve from the data presented in (C). EC50 for each RPE line is shown in the table below the graph.

(E and F) Increasing concentrations of RM, in the presence of FITC labeled POS for 3 h, to hESC-RPE and EX2-RPE. At 3 h, cells were washed and kept with either PBS to monitor total POS (C) or trypan blue to bleach bound POS and monitor internalized POS only (D) with confocal fluorescence microscopy. Higher concentrations of RM increase internalized POS/cell count in both lines. Data are represented as the mean ± SD. N = 3 RPE differentiation rounds (two wells per batch).

Hits Confirmation

For confirming the obtained hits, compounds were reordered from commercial suppliers and added to the cells with and without POS with three different concentrations (5 μM, 10 μM, 15 μM) in 384-well plates (confirmation I) and then in transwell plates (confirmation II). Confirmed hits were subjected to further validation using secondary assays detailed in Supplemental Information.

Data and Code Availability

RNA-seq data were deposited in GEO (https://www.ncbi.nlm.nih.gov/geo/). Reference number: GSE132828.

Study Approval

Permission to work with hESCs was granted by the Robert Koch Institute, Berlin, Germany (license number AZ 3.04.02/0103-A01). The fibroblasts were isolated under full patient consent and approved by Columbia University under institutional review board protocol number AAAR0284. All procedures were in accordance with the Declaration of Helsinki and its amendments.

SUPPLEMENTAL INFORMATION

Supplemental Information can be found online at https://doi.org/10.1016/j.stemcr.2020.10.013.

AUTHOR CONTRIBUTIONS

Investigation and methodology: S.S., K.V., and S.A. designed and performed most of the experiments. R.B. participated in the setup and performance of experiments at the Technology Development Studio facility. S.T. provided MERTK patient fibroblasts. Conceptualization, funding acquisition, and supervision: S.A., M.A., M.K., and E.T. Project administration: S.A. Writing – Original Draft: S.A. Writing – Review & Editing: S.C., K.V., R.B., S.A., M.K., and E.T.

ACKNOWLEDGMENTS

We would like to acknowledge the Federal Ministry of Education and Research (BMBF) for funding this project under the VIP+ program (project name and number: Cleansight, 03VP01220), and the TG70 grant by the SMWK, state of Saxony. M.K. and E.T. were supported by DFG, Cluster of Excellence (CRTD), TU Dresden CRTD; and M.K. by DZNE Helmholtz and HGF ExNet-0007-Phase2. We appreciate the support in cell culture from other members of the Cleansight team, especially Susanne Luft (MSc) and Dr. Dominic Eberle. Additionally, we would like to acknowledge the core facilities of the CMCB Technology Platform, especially advanced imaging, deep sequencing, flow cytometry, and stem cell engineering facilities for providing high-end services.

Received: August 21, 2020
Revised: October 28, 2020
Accepted: October 30, 2020
Published: November 25, 2020

REFERENCES

Almedawar, S., Vafia, K., Schreiter, S., Neumann, K., Khattak, S., Kurth, T., Ader, M., Karl, M.O., Tsang, S.H., and Tanaka, E.M. (2020). MERTK-dependent ensheathment of photoreceptor outer segments by human pluripotent stem cell-derived retinal pigment epithelium. Stem Cell Rep. *14*, 374–389.

Berthold, M.R., Cebron, N., Dill, F., Gabriel, T.R., Kötter, T., Meinl, T., Ohl, P., Sieb, C., Thiel, K., and Wiswedel, B. (2009). KNIME - the Konstanz information miner. ACM SIGKDD Explor. Newsl. *11*, 26.

Brockschnieder, D., Sabanay, H., Riethmacher, D., and Peles, E. (2006). Ermin, a myelinating oligodendrocyte-specific protein that regulates cell morphology. J. Neurosci. *26*, 757–762.

Cai, H., Gong, J., Abriola, L., Hoyer, D., Nyscf Global Stem Cell Array Team, Noggle, S., Paull, D., Del Priore, L.V., and Fields, M.A. (2019). High-throughput screening identifies compounds that protect RPE cells from physiological stressors present in AMD. Exp. Eye Res. *185*. https://doi.org/10.1016/j.exer.2019.04.009.

Eid, B.G., and Gurney, A.M. (2018). Zinc pyrithione activates K+ channels and hyperpolarizes the membrane of rat pulmonary artery smooth muscle cells. PLoS One *13*, 1–13.

Fang, X., Tiyanont, K., Zhang, Y., Wanner, J., Boger, D., and Walker, S. (2006). The mechanism of action of ramoplanin and enduracidin. Mol. Biosyst. *2*, 69–76.

Finnemann, S.C., and Silverstein, R.L. (2001). Differential roles of CD36 and alphavbeta5 integrin in photoreceptor phagocytosis by the retinal pigment epithelium. J. Exp. Med. *194*, 1289–1298.

Fryer, R.M., Muthukumarana, A., Harrison, P.C., Nodop Mazurek, S., Chen, R.R., Harrington, K.E., Dinallo, R.M., Horan, J.C., Patnaude, L., Modis, L.K., and Reinhart, G.A. (2012). The clinically-tested S1P receptor agonists, FTY720 and BAF312, demonstrate subtype-specific bradycardia (S1P1) and hypertension (S1P3) in rat. PLoS One *7*, 4–12.

Golestaneh, N., Chu, Y., Xiao, Y.Y., Stoleru, G.L., and Theos, A.C. (2017). 'Dysfunctional autophagy in RPE, a contributing factor in age-related macular degeneration. Cell Death Dis. *8*. https://doi.org/10.1038/cddis.2016.453.

Hall, M.O., Prieto, A.L., Obin, M.S., Abrams, T.A., Burgess, B.L., Heeb, M.J., and Agnew, B.J. (2001). Outer segment phagocytosis by cultured retinal pigment epithelial cells requires Gas6. Exp. Eye Res. *73*, 509–520.

Inana, G., Murat, C., An, W., Yao, X., Harris, I.R., and Cao, J. (2018). RPE phagocytic function declines in age-related macular degeneration and is rescued by human umbilical tissue derived cells. J. Transl. Med. *16*, 1–15.

Iversen, P.W., Beck, B., Chen, Y.-F., Dere, W., Devanarayan, V., Eastwood, B.J., Farmen, M.W., Iturria, S.J., Montrose, C., Moore, R.A., et al. (2012). HTS assay validation. In Assay Guidance Manual, S. Markossian, G.S. Sittampalam, A. Grossman, K. Brimacombe, M. Arkin, D. Auld, C.P. Austin, J. Baell, J.M.M. Caaveiro, and T.D.Y. Chung, et al., eds. (Bethesda (MD): Company and the National Center for Advancing Translational Sciences), pp. 1089–1113.

Kinch, M.S., Haynesworth, A., Kinch, S.L., and Hoyer, D. (2014). An overview of FDA-approved new molecular entities: 1827-2013. Drug Discov. Today *19*, 1033–1039.

Klionsky, D.J., Abdelmohsen, K., Abe, A., Abedin, M.J., Abeliovich, H., Acevedo Arozena, A., Adachi, H., Adams, C.M., Adams, P.D., Adeli, K., et al. (2016). Guidelines for the use and interpretation

of assays for monitoring autophagy (3rd edition). Autophagy *12*, 1–222.

Lamprecht, M.R., Sabatini, D.M., and Carpenter, A.E. (2007). CellProfiler: free, versatile software for automated biological image analysis. BioTechniques *42*, 71–75.

Law, A.L., Parinot, C., Chatagnon, J., Gravez, B., Sahel, J.A., Bhattacharya, S.S., and Nandrot, E.F. (2015). Cleavage of mer tyrosine kinase (MerTK) from the cell surface contributes to the regulation of retinal phagocytosis. J. Biol. Chem. *290*, 4941–4952.

Liang, F., Hwang, J.H., Tang, N.W., and Hunziker, W. (2018). Juxtanodin in retinal pigment epithelial cells: expression and biological activities in regulating cell morphology and actin cytoskeleton organization. J. Comp. Neurol. *526*, 205–215.

Malo, N., Hanley, J.A., Cerquozzi, S., Pelletier, J., and Nadon, R. (2006). Statistical practice in high-throughput screening data analysis. Nat. Biotechnol. *24*, 167–175.

Miranda, G.E., Abrahan, C.E., Politi, L.E., and Rotstein, N.P. (2009). Sphingosine-1-phosphate is a key regulator of proliferation and differentiation in retina photoreceptors. Invest. Ophthalmol. Vis. Sci. *50*, 4416–4428.

Müller, C., Más Gómez, N., Ruth, P., and Strauss, O. (2014). CaV1.3 L-type channels, maxiK Ca2+-dependent K+ channels and bestrophin-1 regulate rhythmic photoreceptor outer segment phagocytosis by retinal pigment epithelial cells. Cell Signal. *26*, 968–978.

Müller, C., Charniga, C., Temple, S., and Finnemann, S.C. (2018). Quantified F-actin morphology is predictive of phagocytic capacity of stem cell-derived retinal pigment epithelium. Stem Cell Rep. *10*, 1075–1087.

Nandrot, E.F., Anand, M., Almeida, D., Atabai, K., Sheppard, D., and Finnemann, S.C. (2007). Essential role for MFG-E8 as ligand for alphavbeta5 integrin in diurnal retinal phagocytosis. Proc. Natl. Acad. Sci. U S A *104*, 12005–12010.

Naska, S., Yuzwa, S.A., Johnston, A.P., Paul, S., Smith, K.M., Paris, M., Sefton, M.V., Datti, A., Miller, F.D., and Kaplan, D.R. (2016). 'Identification of drugs that regulate dermal stem cells and enhance skin repair. Stem Cell Rep. *6*, 74–84.

Niemann, S., Li, Y., Thompson, D.A., Weir, J., Orth, U., Jacobson, S.G., Apfelstedt-Sylla, E., and Vollrath, D. (2000). Mutations in MERTK , the human orthologue of the RCS rat retinal dystrophy gene , cause retinitis pigmentosa. Nat. Genet. *26*, 270–271.

Priest, B.T., and Erdemli, G. (2014). Phenotypic screening in the 21st century. Front. Pharmacol. https://doi.org/10.3389/fphar.2014.00264.

Reeder, N.L., Xu, J., Youngquist, R.S., Schwartz, J.R., Rust, R.C., and Saunders, C.W. (2011). The antifungal mechanism of action of zinc pyrithione. Br. J. Dermatol. *165*, 9–12.

Schwartz, J.R. (2016). Zinc pyrithione: a topical antimicrobial with complex pharmaceutics. J. Drugs Dermatol. *15*, 140–144.

Strauss, O. (2005). The retinal pigment epithelium in visual function. Physiol. Rev. *85*, 845–881.

Swinney, D.C. (2013). Phenotypic vs. target-based drug discovery for first-in-class medicines. Clin. Pharmacol. Ther. *93*, 299–301.

Tang, J., Tang, J., Ling, E.A., Wu, Y., and Liang, F. (2009). Juxtanodin in the rat olfactory epithelium: specific expression in sustentacular cells and preferential subcellular positioning at the apical junctional belt. Neuroscience *161*, 249–258.

Thokala, S., Inapurapu, S., Bodiga, V.L., Vemuri, P.K., and Bodiga, S. (2017). Loss of ErbB2-PI3K/Akt signaling prevents zinc pyrithione-induced cardioprotection during ischemia/reperfusion. Biomed. Pharmacother. *88*, 309–324.

Valentine, W.J., Kiss, G.N., Liu, J., E.S., Gotoh, M., Murakami-Murofushi, K., Pham, T.C., Baker, D.L., Parrill, A.L., Lu, X., et al. (2010). (S)-FTY720-Vinylphosphonate, an analogue of the immunosuppressive agent FTY720, is a pan-antagonist of sphingosine 1-phosphate GPCR signaling and inhibits autotaxin activity. Cell Signal. *22*, 1543–1553.

Vestri, A., Pierucci, F., Frati, A., Monaco, L., and Meacci, E. (2017). Sphingosine 1-phosphate receptors: Do they have a therapeutic potential in cardiac fibrosis? Front. Pharmacol. *8*. https://doi.org/10.3389/fphar.2017.00296.

Vishwanathan, R., Chung, M., and Johnson, E.J. (2013). A systematic review on zinc for the prevention and treatment of age-related macular degeneration. Invest. Ophthalmol. Vis. Sci. *54*, 3985–3998.

Wang, J.Z., Xiao, N., Zhang, Y.Z., Zhao, C.X., Guo, X.H., and Lu, L.M. (2016). Mfsd2a-based pharmacological strategies for drug delivery across the blood-brain barrier. Pharmacol. Res. *104*, 124–131.

Wimmers, S., Karl, M.O., and Strauss, O. (2007). Ion channels in the RPE. Prog. Retin. Eye Res. *26*, 263–301.

Wong, B.H., Chan, J.P., Cazenave-Gassiot, A., Poh, R.W., Foo, J.C., Galam, D.L., Ghosh, S., Nguyen, L.N., Barathi, V.A., Yeo, S.W., et al. (2016). 'Mfsd2a is a transporter for the essential ω -3 fatty acid docosahexaenoic acid (DHA) in eye and is important for photoreceptor cell. Development *291*, 10501–10514.

Zhu, Y., Carido, M., Meinhardt, A., Kurth, T., Karl, M.O., Ader, M., and Tanaka, E.M. (2013). Three-dimensional neuroepithelial culture from human embryonic stem cells and its use for quantitative conversion to retinal pigment epithelium. PLoS One *8*, e54552.

Zorych, K., Sinha, D., Gómez, N.M., Zorych, K., Dutrow, E.V., Dhingra, A., Mullins, R.F., Stone, E.M., Gamm, D.M., Boesze-Battaglia, K., and Aguirre, G.D. (2017). 'Bestrophinopathy: an RPE-photoreceptor interface disease'. Prog. Retin. Eye Res. *58*, 70–88.

Stem Cell Reports, Volume *15*

Supplemental Information

A Human Retinal Pigment Epithelium-Based Screening Platform Reveals Inducers of Photoreceptor Outer Segments Phagocytosis

Sven Schreiter, Katerina Vafia, Rico Barsacchi, Stephen H. Tsang, Marc Bickle, Marius Ader, Mike O. Karl, Elly M. Tanaka, and Seba Almedawar

Supplemental Information: Figures and Legends

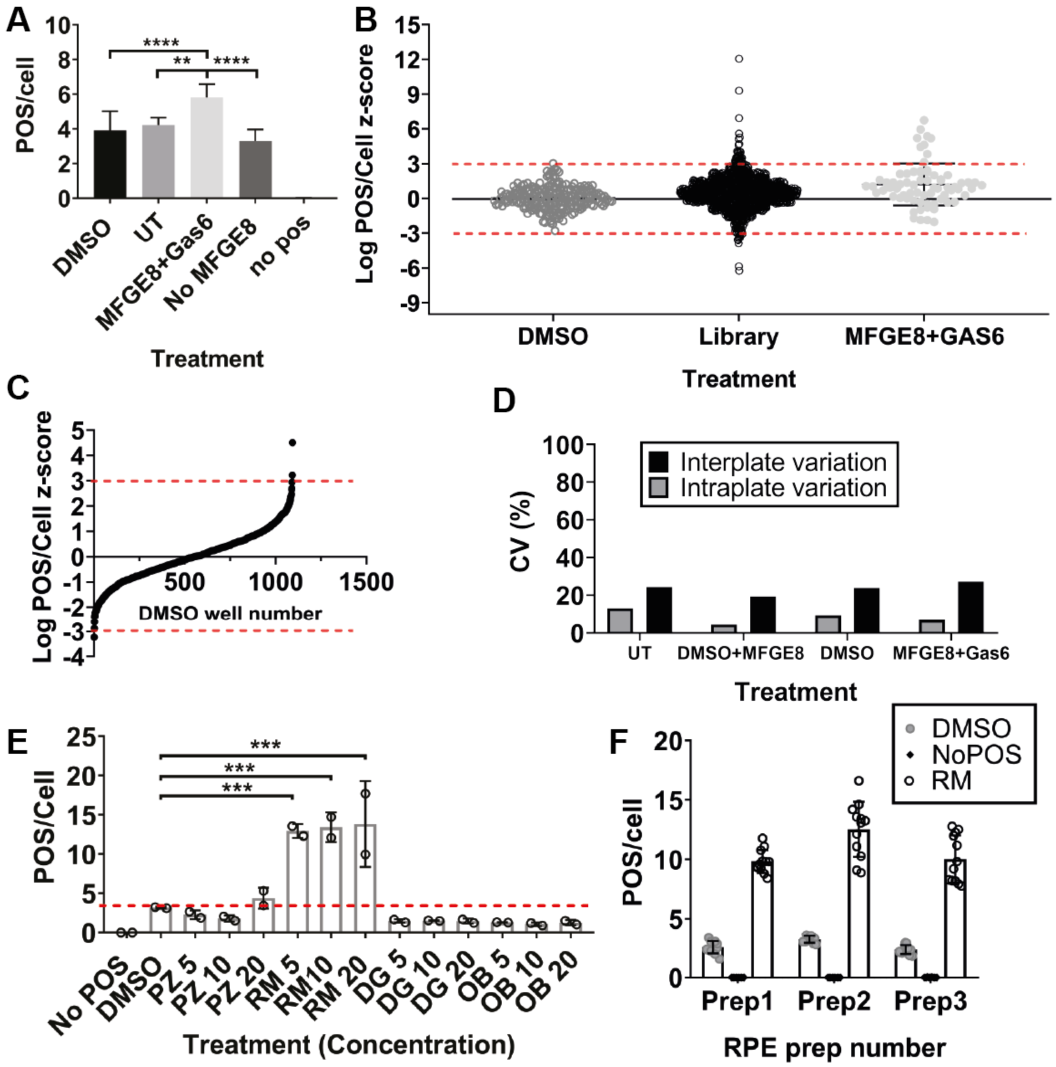

Figure S1. The screening platform demonstrates low false positive/negative rate and reproducibility across different human RPE preps. Related to Figure 3.

(A) Controls from one screening plate. MFGE8/GAS6: 2.5 µg/ml were added from each ligand. UT: Untreated. Data are represented as the mean ± SD. n=24 wells DMSO and n=4 wells of each treatment. Significance was calculated using one-way-ANOVA. All treatments were compared to DMSO. ns >0.05, *<0.05, ** <0.01, ***<0.001, ****<0.0001.

(B) The z-score of the log of the POS/cell within each DMSO, Library, or MFGE8+GAS6 well that was used in the screen was plotted. Dotted lines show the z-score threshold (-3, +3) that was used in the screen to determine hits. The graph shows that the library is spread over the z-score axis, while DMSO wells are concentrated between -3 and +3, and most of the MFGE8+GAS6 wells show a positive z-score.

(C) The z-score of the log of the POS/cell within each DMSO well that was used in the screen was plotted. The graph shows two wells that were identified as false positive and 1 that was identified as false negative.

(D) Interplate (all screening plates from 4 runs) and Intraplate (plate 1 from run 1) variation of the control wells used in the screen.

(E) Confirmation in transwells (Step III). Related to **Table S5**. Data are represented as the mean ± SD. N=2 biological repeats. Significance was calculated using one-way-ANOVA. All treatments were compared to DMSO. ns >0.05, *<0.05, ** <0.01, ***<0.001, ****<0.0001.

(F) Increase of POS phagocytosis using RM (RM) compared to DMSO was confirmed over 3 RPE differentiation rounds performed on different days. Data represent the mean of the total POS/cell count ± SD. n=12 wells per RPE differentiation batch.

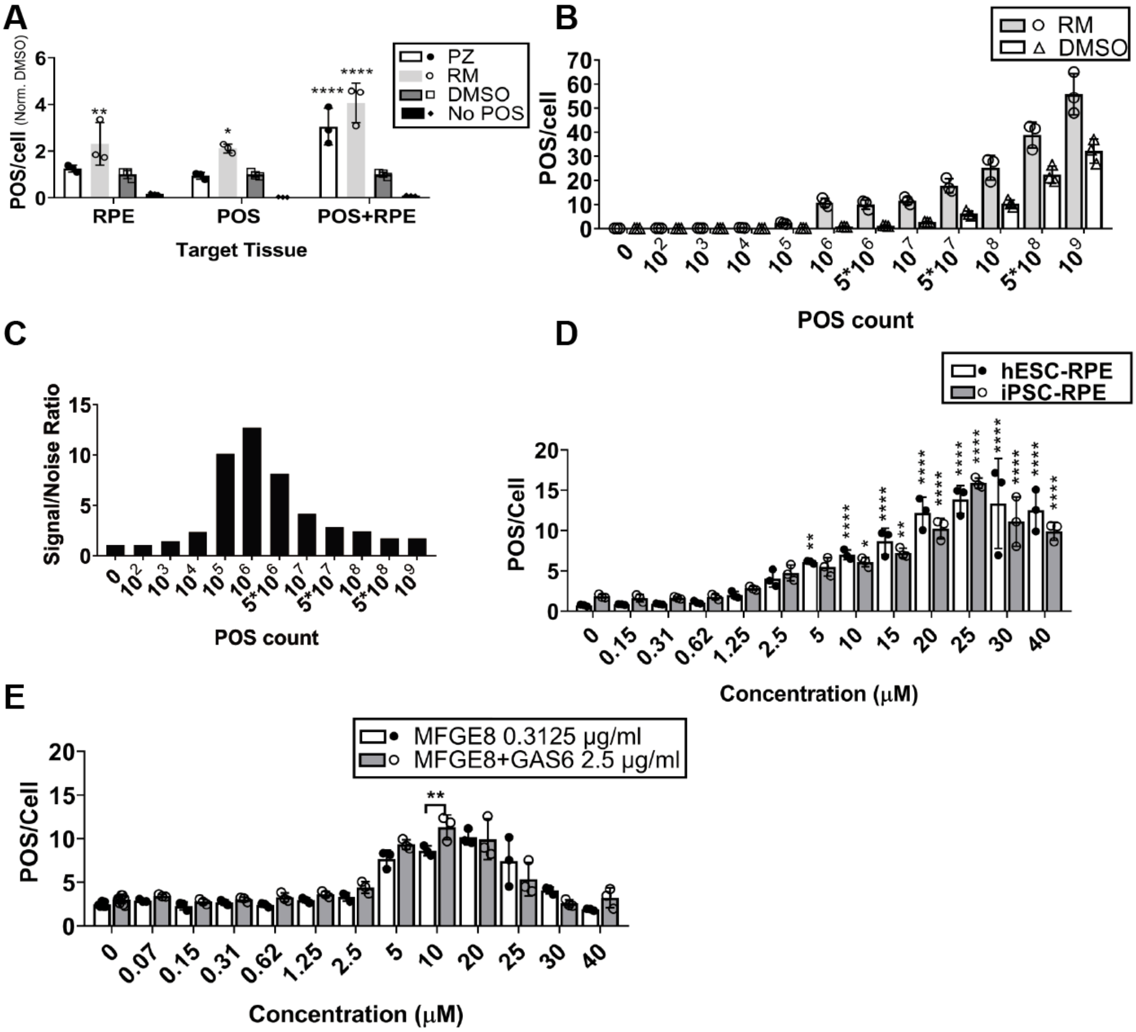

Figure S2. Orthogonal assays show that Ramoplanin acts on both photoreceptor and human RPE cells in a dose dependent manner. Related to Figure 3.

(A) Ramoplanin (RM)/Pirithione zinc (PZ)/ DMSO were added to hESC-RPE for 1 hour then they were washed off before addition of POS (Target Tissue: RPE), or added to AF555-labeled POS for 1 hour and washed off before addition of POS to RPE (Target Tissue: POS). Alternatively, AF555-labeled POS were added to the cells which were previously treated with the compounds without a washing step in between (Target Tissue: POS+RPE). RM seems to act on both POS and RPE. Data are normalized to DMSO in each target tissue separately and are represented as the mean ± SD. N=3 RPE differentiation batches. Significance was calculated using two-way-ANOVA. Samples of the same target tissue were compared to DMSO. ns >0.05, *<0.05, ** <0.01, ***<0.001, ****<0.0001.

(B) RM was added to RPE cells in the presence of different concentrations of AF555-labeled POS for 3 hours. Fixed cells were analyzed with confocal fluorescence microscopy. Addition of higher concentrations led to increase in the POS/cell count in both RM and DMSO. Data are represented as the mean ± SD. N=3 RPE differentiation batches.

(C) Ratio of the POS/cell count in RM treated samples to DMSO samples. The best signal to noise ratio was obtained when cells were challenged with 10^6 POS/well.

(D) Increasing concentrations of RM were added to RPE cells differentiated from hESC and iPSC in the presence of AF555-labeled POS for 3 hours. Fixed cells were analyzed with confocal fluorescence microscopy. Higher concentrations of RM increase the POS/cell count up to 25 μM. Addition of higher concentrations causes a drop in the POS/cell count. Data are represented as the mean ± SD. N=3 RPE differentiation batches. Significance was calculated using two-way-ANOVA. All treatments were compared to DMSO. ns >0.05, *<0.05, ** <0.01, ***<0.001, ****<0.0001.

(E) Increasing concentrations of RM were added to hESC-RPE in the presence of AF555-labeled POS and low MFGE8 concentration (0.3125 μg/ml) or MFGE8 and GAS6 (each at 2.5 μg/ml) for 3 hours. An additive effect is seen and is significant at 10 μM concentration of RM. Data are represented as the mean ± SD. N=3 RPE differentiation batches. Significance was calculated using two-way-ANOVA. Samples of the same RM concentration, but different ligands concentration were compared. ns >0.05, *<0.05, ** <0.01, ***<0.001, ****<0.0001.

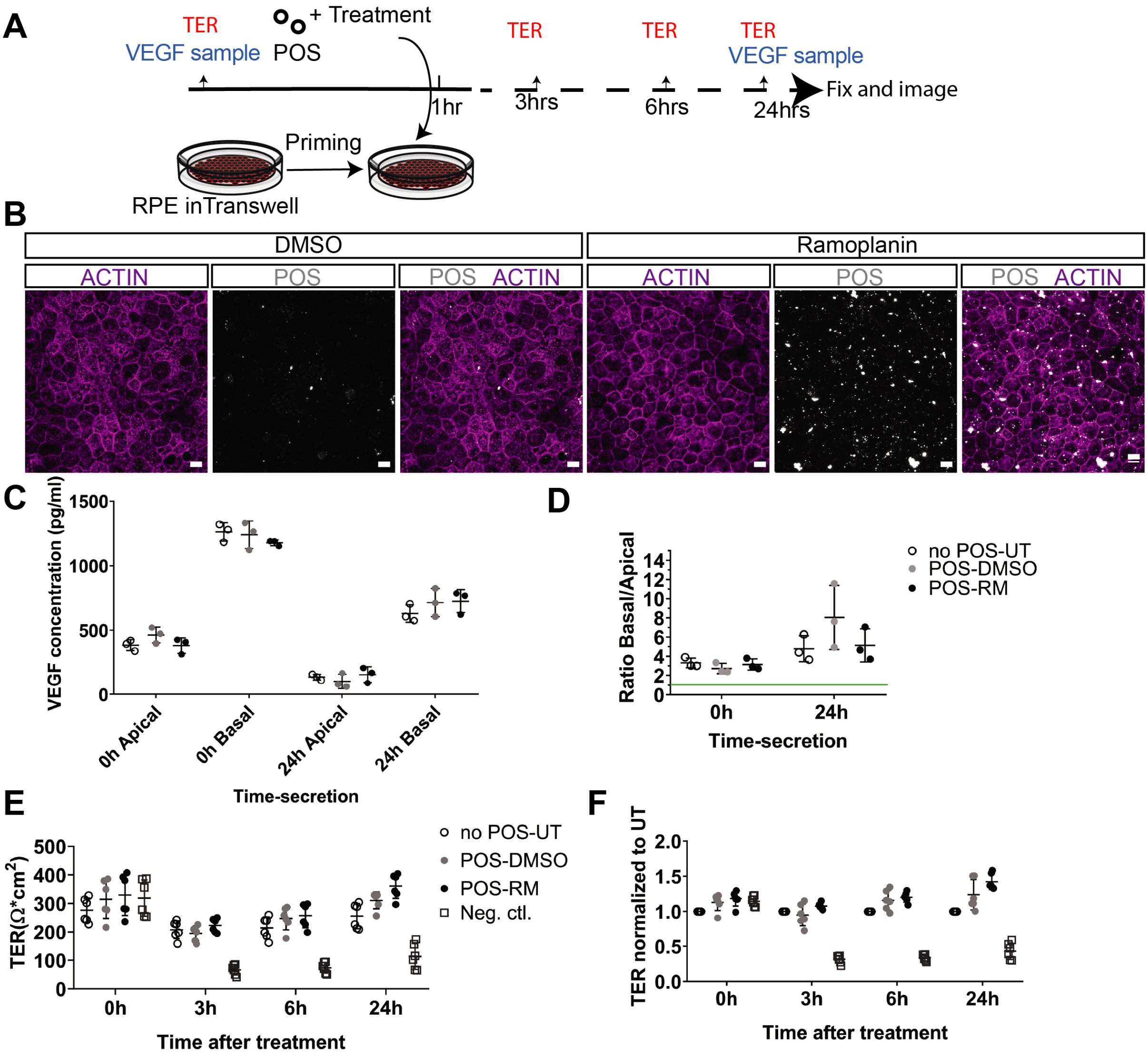

Figure S3. Ramoplanin does not affect human RPE polarized secretion of VEGF and RPE monolayer integrity. Related to Figure 4.

(A) Experiment pipeline.

(B) Fluorescence images of hESC-RPE cells cultured in transwells challenged with AF555-labeled POS (Gray) and Ramoplanin (RM)/DMSO for 3 hours. Fixed cells were labeled with Phalloidin (Magenta) to mark the boarders of the cells. Scale bar: 10 µm.

(C) VEGF values before and 24 hours after treatment with RM/DMSO and POS. Data are represented as the mean ± SD. N=3 RPE differentiation batches. RM does not compromise the polarized secretion of VEGF after 24 hours of treatment. UT: untreated.

(D) Ratio of the concentration of VEGF in the basal side of the RPE cells to the apical side. Data are represented as the mean ± SD. N=3 RPE differentiation batches.

(E) Transepithelial resistance (TER) values of RPE cells before and 3, 6, 24 hours after treatment with RM/DMSO and POS. A scratch was applied in the middle of the transwell to disturb the monolayer integrity (Neg. ctl.). Data are represented as the mean ± SD. N=3 RPE differentiation batches. RM does not compromise the TER of the cells after 24 hours of treatment.

(F) TER values were normalized to DMSO at each time point and plotted. Data are represented as the mean ± SD. N=3 RPE differentiation batches.

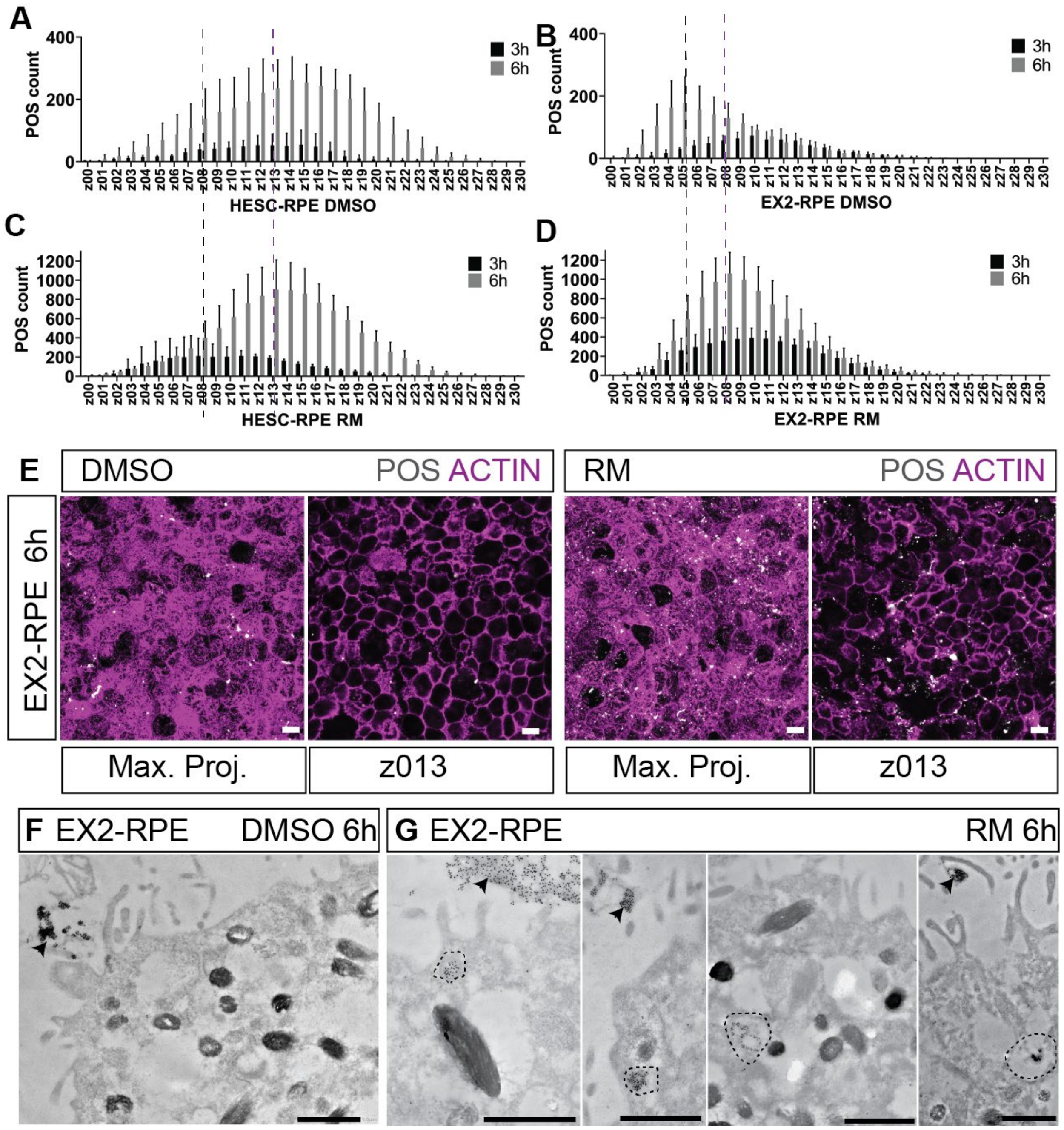

Figure S4. Ramoplanin rescues internalization defect in human MERTK mutant RPE determined by means of fluorescence confocal microscopy and transmission electron microscopy (TEM). Related to Figure 7.

(A-D) AF555- labeled POS were added to wild-type RPE (hESC-RPE) and MERTK mutant RPE (EX2-RPE) and left for 3 hours and 6 hours after POS addition. Cells were washed, fixed and labeled with phalloidin to mark the apical and the basal side of the cells. Confocal fluorescence microscopy images were acquired with 31 optical sections (z-stacks) and analyzed to obtain POS count. z0 is at the apical side of the RPE z30 is at the basal side of the RPE. Data represent the mean of the total POS count ± SD. N=3 RPE differentiation batches. In hESC-RPE POS are internalized after 6 hours and are mostly found in z13 (A). The amount of POS in z13 increases after treatment with Ramoplanin (RM) (C). At 6 hours POS still accumulate at the surface of the Ex2-RPE (z05, black dotted line, B), while in the presence of RM POS move towards the inside of the cell (z08, purple dotted line, D).

(E) Fluorescence microscopy images of EX2-RPE at 6 hours after addition of AF555-labeled POS and RM/DMSO as in **(B-D)**. Actin is labeled with phalloidin (Magenta). Maximum intensity projection of the 31 optical sections and z13 only are shown. The number of internalized POS is higher in z13 in RM treated samples compared to DMSO.

(F-G) Transmission electron microscopy of EX2-RPE cells treated with POS and DMSO/RM for 6 hours. In DMSO samples, POS are mostly found on the surface of the RPE, while in RM samples internalized POS at the apical side of the RPE could be detected. Scale bar: 1 µm.

Table S1. List of compounds in the FDA approved library (Excel Table). Related to Figure 3.

Table S2. List of hits from the primary screening assay. Related to Figure 3.

Compound name	Mean POS/cell DMSO	SD of the Mean POS/cell DMSO	POS/cell Hit	Z-score	Structure
**CARBIDOPA**	7.72627946	1.80183412	18.18239142	3.859843239	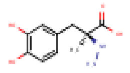
	3.97027337	0.72160097	6.987130292	3.293712615	
**RAMOPLANIN**	4.00344939	0.77791355	10.65762809	4.845892949	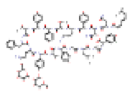
	4.31512749	0.62271494	8.368666042	9.050912693	
	6.1640321	1.53512574	19.56056543	4.942319314	
	3.536627	0.44530994	10.72073678	5.00557346	
**CEPHAPIRIN SODIUM**	4.02359632	0.61315362	6.35266788	3.007959189	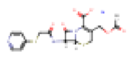
	6.24270555	1.32986439	13.73751677	3.388429658	
**DOXORUBICIN**	5.59807957	1.23901679	27.5891581	3.388646968	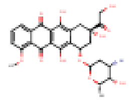
	4.73843874	1.26001387	11.65284639	7.655831648	
**HOMIDIUM BROMIDE**	3.3279917	0.66323947	39.13526909	13.12391821	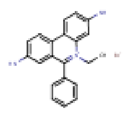
	3.38773503	0.74357468	53.79315086	11.13684767	
	4.42129613	0.74605771	96.48429397	18.78772985	
	6.69223467	1.3533011	110.1212104	14.40519773	
**MERBROMIN**	3.3279917	0.66323947	9.605061573	5.697155474	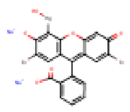
	3.38773503	0.74357468	16.28800234	6.371631183	
	10.8106779	1.49126386	16.77847338	3.185274372	
	6.69223467	1.3533011	15.69051785	4.449296127	
**PYRITHIONE ZINC**	4.42129613	0.74605771	8.062179186	3.725484414	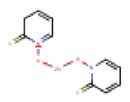
	10.8106779	1.49126386	20.57312327	4.631893696	
	6.69223467	1.3533011	18.92793233	5.407738765	
**SUCCINYLSULFATHIAZOLE**	1.76719904	0.19447114	2.795	4.406777297	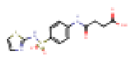
	7.18489185	1.19956523	12.08333333	3.286567331	
**DIGOXIN**	4.02359632	0.61315362	1.969020291	-4.518464121	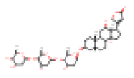
	6.24270555	1.32986439	2.655620834	-3.452450781	
**OUABAIN**	6.29444936	1.21391753	3.408987603	-3.68263515	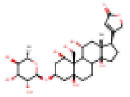
	5.93189068	1.51873745	2.351151505	-3.152237244	
**POTASSIUM p-AMINOBENZOATE**	4.31512749	0.62271494	1.842309897	-5.477229687	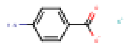
	4.00344939	0.77791355	1.270037248	-6.755468742	
	3.536627	0.44530994	1.525836523	-6.196451042	
**SENNOSIDE A**	3.50911	0.69084309	1.142751273	-5.504344515	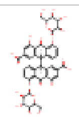
	6.42701984	1.32842536	2.999972133	-3.434578951	

Table S3. Autofluorescence values of hits in the absence of POS (Excel Table). Related to Figure 3.

Table S4. List of hits from confirmation I in 384-wells plates. Related to Figure 3.

Treatments	POS per Cell Mean	POS per Cell (Standard deviation)	z-score
**DMSO 0.2%**	8.150224049	1.931809451	
**No POS**	0.010885128	0.003674366	
**Digoxin 5**	4.419513352	0.934296173	-2.59094
**Digoxin 10**	4.536797936	1.368324695	-2.76922
**Digoxin 20**	5.18449646	0.988038768	-1.76115
**Ouabain 5**	4.594461362	1.3591871	-2.44608
**Ouabain 10**	4.509466427	1.841329131	-2.68072
**Ouabain 20**	5.62181552	0.988865715	-1.37035
**Proscillaridin 5**	4.700784182	1.53164408	-2.77648
**Proscillaridin 10**	4.725888169	1.694488286	-2.61604
**Proscillaridin 20**	5.453510783	0.950739994	-1.58906
**Pyrithione Zinc 5**	7.770393878	1.628158803	-0.27707
**Pyrithione Zinc 10**	8.762359994	2.009086981	0.490385
**Pyrithione Zinc 20**	20.89329247	1.723136342	4.360371
**Ramoplanin 5**	16.75894992	3.420217172	3.222757
**Ramoplanin 10**	19.17718295	2.73164563	4.030754
**Ramoplanin 20**	23.35658231	5.046969091	4.826715
**Thiostrepton 5**	8.94419779	1.348363893	0.46252
**Thiostrepton 10**	8.910353091	1.231485311	1.458945
**Thiostrepton 20**	10.82155098	2.236051978	1.429046

Table S5. List of hits from confirmation II in transwells. Related to Figure S1.

Treatment	POS/Cell	Median between fields	Mean between wells
**DMSO**	3.08	3.114	3.189
	5.168		
	3.148		
	2.212		
	3.272	3.264	
	3.66		
	3.096		
	3.256		
**Ouabain 5**	1.372	1.332	1.306
	1.2		
	1.292		
	1.66		
	1.292	1.28	
	1.4		
	1.268		
	1.164		
**Ouabain 10**	0.6	0.914	1.068
	1.084		
	0.744		
	1.172		
	2.504	1.222	
	1.304		
	1.14		
	0.852		
	1.416	1.508	1.266

**Ouabain 20**	1.468		
	1.872		
	1.548		
	1.016	1.024	
	0.96		
	1.324		
	1.032		
**Pyrithione Zinc 5**	2.872	2.684	2.284
	2.496		
	3.184		
	1.724		
	1.568	1.884	
	2.084		
	2.008		
	1.76		
**Pyrithione Zinc 10**	1.864	2.076	1.801
	4.116		
	2.288		
	1.448		
	1.756	1.526	
	1.728		
	1.208		
	1.324		
**Pyrithione Zinc 20**	3.368	3.448	4.396
	5.044		
	3.528		
	2.548		
	4.66	5.344	
	6.028		
	4.38		
	7.476		
**Ramoplanin 5**	9.392	13.564	12.939
	15.204		
	13.804		
	13.324		
	13.576	12.314	
	11.772		
	12.856		
	8.92		
**Ramoplanin 10**	16.588	14.744	13.415
	8.316		
	14.976		
	14.512		
	12.284	12.086	
	11.888		
	9.584		
	14.552		

**Ramoplanin 20**	8.268	9.966	13.843
	11.664		
	4.772		
	13.04		
	25.848	17.72	
	22.092		
	9.38		
	13.348		
**Digoxin 5**	1.4	1.312	1.484
	1.244		
	1.344		
	1.28		
	2.432	1.656	
	1.224		
	1.248		
	2.064		
**Digoxin 10**	1.724	1.548	1.523
	1.428		
	1.184		
	1.668		
	0.968	1.498	
	0.86		
	2.028		
	2.048		
**Digoxin 20**	2.12	1.684	1.464
	2.684		
	1.248		
	0.948		
	0.86	1.244	
	1.508		
	0.98		
	1.568		
**No POS**	0	0	0
	0		
	0		
	0		
	0	0	
	0.004		
	0		
	0		

Table S6. RNA seq data of samples treated with POS and Ramoplanin compared to samples treated with POS and DMSO (Excel Table). Related To Figure 5.

Table S7. IPA based analysis of RNA seq data (Excel Table). Related to Figure 5.

Table S8. Resources Table

Reagent or Resource	SOURCE	IDENTIFIER
**Antibodies**		
rhodopsin (TEM)	Sigma	O4886
rhodopsin (Immuno-blot)	Abcam	ab5417
ZO1	Invitrogen	402200,
Bestrophin1	Abcam	ab2182
MITF	Abcam	ab122982
MERTK	Abcam	ab52968
EZRIN	Santa Cruz	sc-58758
β-TUBULIN	Thermo Fisher	PA5-16863
Alexa Fluor 488 rabbit	Invitrogen	A21206
Alexa Fluor 488 mouse	Invitrogen	A21202
Alexa Fluor 647 rabbit	Invitrogen	A31573
Alexa Fluor 647 mouse	Invitrogen	A31571
HRP-rabbit secondary antibody	Invitrogen	32260
HRP- mouse secondary antibody	Thermo Fisher	62-6520
rabbit anti mouse bridging antibody	Sigma	M7023_2ML
protein A gold	CMC Utrecht	https://www.cellbiology-utrecht.nl/products.html
**Chemicals, Peptides, and Recombinant Proteins**		
soybean trypsin inhibitor	Sigma	T6522
trypsin-EDTA	Gibco	T3924
ACTIVIN A	R&D	338-AC-050
Antibiotic-Antimycotic	Gibco	15240-062
TrypLE	Thermo Fisher	12563011
protease inhibitors	Roche	4693132001
Fluorescein FITC	Invitrogen	F2182
Alexa Fluor 555	Invitrogen	A20009
Alexa Fluor 647 Phalloidin	Invitrogen	A22287
GAS6	R&D systems	885-GSB
MFGE8	R&D systems	2767-MF
FTY720	SIGMA	SML0700
Tunicamycine	SIGMA	T7765
Serum (FBS)	gibco	26140-079
trypan blue	Sigma	T8154
Paraformaldehyde	Science Services	E15714-S
Glutaraldehyde	Science Services	E16220
Hoechst 33342	Thermo Fisher	H1399
RIPA	Thermo Fisher	89900
BSA	SERVA	11926.03
10% Mini-PROTEAN® TGX™ Precast Protein Gels	BioRad	4561033
Precision Plus Protein™ WesternC™	BioRad	1610376
SuperSignal™ West	Thermo Fisher	34075
Precision Protein™ StrepTactin-HRP Conjugate	BioRad	1610380
Lowicryl K4M resin	Science Services	14330
osmium tetroxide	Science Services	19190

mTESR1	StemCell Technologies	85870
Dispase	StemCell Technologies	7923
Rock inhibitor Y-27632	StemCell Technologies	78003
**Critical Commercial Assays**		
RNeasy Mini kit	Qiagen	74106
SuperScript™ II Reverse Transcriptase	Invitrogen	18064022
SYBR™ Green PCR Master Mix	Applied Biosystems	4309155
NEBNext Poly(A) mRNA Magnetic Isolation Module	NEB	E7490L
NEBNext Ultra Directional RNA Library Prep Kit for Illumina	NEB	E7420L
XP bead purification	Beckman Coulter	A63882
VEGF Human ELISA Kit	Invitrogen	KHG0112
Qubit dsDNA HS Assay Kit	Invitrogen	Q32854
H9 human embryonic stem cell line	WiCell	WA-09
MERTK knockout in H9 (MERTK-EX2)	Previous study	DOI: 10.1016/j.stemcr.2020.02.004
MERTK knockout in H9 (MERTK-EX14)	Previous study	DOI: 10.1016/j.stemcr.2020.02.004
RP38 patient iPSC (MERTK)	Previous study	DOI: 10.1016/j.stemcr.2020.02.004
TSS isogenic iPSC control	Previous study	DOI: 10.1016/j.stemcr.2020.02.004
GraphPad Prism	La Jolla California USA,	www.graphpad.com
Knime	Berthold et al., 2009	
Cell Profiler	Lamprecht et al., 2007	
GSNAP (v2014-12-17)		http://research-pub.gene.com/gmap.
featureCounts (v1.4.6)	Liao et al., 2014	http://subread.sourceforge.net/
R		https://www.r-project.org/
DESeq2 R package (v1.6.3)	Love et al., 2014	https://bioconductor.org/packages/release/bioc/html/DESeq2.html
384 well plates	Greiner	781091
Transwells	Corning	3470
ERMN F:	SIGMA	ATGGGGATAAACCACCTGAAAAC
ERMN R:	SIGMA	GAGCAGCATGTTCCCTTGTAA
S1PR5 F:	SIGMA	GCGCACCTGTCCTGTACTC
S1PR5 R:	SIGMA	GTTGGTGAGCGTGTAGATGATG
MFSD2A F:	SIGMA	ATCAGCACCGAGCAGACTG
MFSD2A R:	SIGMA	GCTATTGAGGTCCTGGAAACAAG
Pharmakon 1600	MircoSource Discovery Systems	
Ramoplanin	SIGMA	R1781
Pyrithion Zinc	SIGMA	PHR1401
Digoxin	SIGMA	1200000
Ouabain	SIGMA	O0200000
Cephapirin Sodium	SIGMA	1102500
Potassium p-Aminobenzoate	SIGMA	A0254
Sennoside A	SIGMA	1612018
Carbidopa	SIGMA	C1335
Doxorubicin	SIGMA	1225703
Homidium Bromide	SIGMA	E8751
Merbromin	SIGMA	M7011
Succinylsulfathiazole	SIGMA	S8752

Supplemental Experimental Procedures:

Cell lines and cell culture

RPE derived from H9 hESC cell line (hESC-RPE) were used as wild-type control. Wild-type iPSC line was derived in collaboration with the stem cell and engineering facility in the CRTD. Fibroblasts were isolated from skin biopsies from RP38 patients and were reprogrammed using the non-integrating Sendai virus. RPE differentiated from patient iPSC is referred to as "MERTK-RPE" throughout the manuscript. *MERTK* gene editing with CRISPR/CAS9 was done in H9 hESC. Two modified hESC lines were obtained EX2 and EX14. Differentiated RPE are referred to as "EX2-RPE" and "EX14-RPE" throughout the manuscript. Detailed description of the generation of *MERTK* knock out cell lines, and patient iPSC line, and the characterization of the pluripotent cell lines were previously described in Almedawar et al. (Almedawar et al., 2020). All RPE cells were differentiated on transwell filters as previously described (Zhu et al., 2014, 2013) with some modifications. Briefly, dissociated stem cell colonies were counted prior to embedding in matrigel and the amount of matrigel was adjusted accordingly. Following neuroepithelial cysts trypsinization between 100,000 and 150,000 cells were plated on transwells in the presence of 5 µM rock inhibitor. Activin A concentration was reduced to 0.02 µg/ml for RPE differentiation from hESC and to 0.01 µg/ml for RPE differentiation from iPSC. RPE cells were passaged for expansion two times on transwells before use. For some experiments and as indicated in the experimental schemes, RPE cells were passaged first on transwells for expansion and then to 384-wells plates and used after 13 days. For passaging, cells on transwells were incubated with trypsin-EDTA (TE) for 10 minutes at 37ºC and 5% CO2. After incubation, cells were vigorously pipetted to obtain single cells, and transferred to a tube containing RPE medium plus soybean trypsin inhibitor. Next, RPE single cells were washed by centrifugation at 180 g for two minutes. Finally, cells were resuspended with RPE media containing activin A and 1x Antibiotic-Antimycotic, and 45,000 cells per one 384 well, or 300,000 cells per transwell were plated.

Immunofluorescence labeling of RPE cells

Cells were fixed with 4% paraformaldehyde (PFA). After several washes with PBS, they were treated with the quenching solution (1x PBS, 100 mM Glycine, 0.3% Triton X-100) for twenty minutes and then were incubated with the blocking solution (1xPBS, 1% BSA, 0.3% Triton X-100) for one hour. The primary and secondary antibodies were diluted in the blocking solution and incubated overnight at 4°C. Cell nuclei were counterstained by addition of Hoechst 33342 (1mg/ml) with the secondary antibody incubation step. The primary antibodies and their working dilutions were as follows: ZO1 (1:200), (1:500), MITF (1:500), MERTK (1:1000). The secondary antibodies Alexa Fluor 488/647 rabbit and mouse were used at 1:500 dilution. Phalloidin 647 was added to the cells after the secondary antibody (1:1000) overnight.

Isolation of porcine POS

POS were isolated from porcine eyes, as described previously (Molday et al., 1987) with some modifications. Briefly, the retinal tissue was isolated from 50 porcine eyes and homogenized in a buffer containing protease inhibitors, 0.2 mM Tris-HCl, 0.1 mM Glucose, 130 mM $NaCl_2$, 0.1 mM Taurine, and 0.02 mM $MgCl_2$. The homogenized retinal tissue was split in 6 tubes containing a sucrose gradient of 27%, 33%, 41%, 50% and 60%, and centrifuged in the Beckmann Coulter ultracentrifuge B409 at 28000 rpm for one hour at 4º C. The orange band was collected in Nalgene centrifuge tubes, and centrifuged at 13000 g for 10 minutes. Finally, the pellets were resuspended with POS storage solution containing protease inhibitors, 10 mM phosphate buffer pH 7.2, 100 mM $NaCl_2$ and 2.5% sucrose and stored at -80 ºC until they were used.

POS labeling

For labeling, Fluorescein (FITC) or Alexa Fluor 555 (AF555) were added to the POS after thawing for 1 hour at 25°C with shaking (500 rpm). Next, POS were centrifuged at 9000 g at 4°C for 10 minutes, and washed twice with the washing buffer, containing: 10% sucrose, 20 mM phosphate buffer pH7.2 and 5 mM Taurine.

Phagocytosis assay

For all experiments, cells were primed with the different treatments for 1 hour before the addition of POS. POS particles were sonicated in 500 µl RPE media containing the different treatments (MFGE8, GAS6) for 5 seconds 10% power with BRANSON Digital Sonifier 450 before addition to the cells to prevent aggregation. The composition of RPE media has been described previously(Zhu et al., 2014, 2013). Before seeding, POS were quantified using the Neubauer chamber combined with fluorescence microscopy imaging and CellProfiler analysis. In all phagocytosis experiments, unbound POS were washed away before fixation, at various time points after POS addition, depending on the purpose of the experiment, using PBS containing 1 mM $MgCl_2$ and 0.2 mM $CaCl_2$ (PBS-MC). GraphPad Prism was used for statistical significance calculations as indicated in the figure legend and final graph presentation.

Primary screening phagocytosis assay

HESC-RPE were plated in 384-wells plates for 13 days before they were used for screening. On the screening day RPE cells were primed for one hour with the library or the control treatment including MFGE8 and GAS6 (2.5 µg/ml) and DMSO only. The compounds were added to the cells in the presence of 25 µl RPE media containing 0.3125 µg/ml MFGE8 using Echo acoustic liquid handler (Beckmann Coulter). During the priming, POS were labeled with AF555 and prepared for addition. After one hour of priming 25 µl of labeled POS were added to the cells and the library compounds were added again, so that the final concentration of the compounds in the library is 10 µM. After three hours incubation at 37ºC, 5% CO2, cells were washed five times with PBS and incubated with PBS with Hoechst overnight. Plates were acquired the next day using Yokogawa's CV7000S confocal microscope. The library consisting of 1600 FDA approved compounds was distributed over nine 384-wells plates with 192 compounds each except for plate 9 which contains 64 compounds.

The screen was performed four times using four consecutive RPE preps. Besides the library, the screening plate consisted of twenty-four wells containing 0.3125 µg/ml MFGE8, DMSO (0.1%) and POS, four wells containing DMSO (0.1 %) and POS only without MFGE8, twelve wells without POS, eight wells MFGE8 and Gas6 (2.5 µg/ml) and POS, and four wells untreated (No DMSO or MFGE8) with POS.

Imaging and Image analysis

During Imaging, nine optical sections from six fields in each well were acquired, using the 63X objective, covering the different areas in the well. Acquired images were imported into the CellProfiler (Lamprecht et al., 2007) software for image analysis. POS and nuclei were segmented and values of the number, size and intensity were obtained.

Data and statistical analysis

The data obtained from image analysis was imported into KNIME (Berthold et al., 2009) for statistical analysis. The median count of the POS between the 6 images was divided by the median count of cells per well to obtain the POS/cell count. To determine positive and negative hits, log POS/cell for each well was calculated and used to calculate the z-score using the following formula:

$$z - score = \frac{Log\frac{POS}{cell}\ treatment - Mean\ Log\frac{POS}{cell}DMSO}{SD\ DMSO}$$

Compounds that gave a z-score of ≥+3 were considered as positive hits and compounds that gave a z-score of ≤-3 were considered negative hits.

To calculate the coefficient of variation (CV), the following formula was used:

$$CV = \frac{Mad\ POS/cell}{Median\ POS/cell}X100$$

Hits confirmation:

For confirming the obtained hits the hits were reordered as powder from Sigma-Adrich and added to the cells with and without POS with three different concentrations (5 µM, 10 µM, 15 µM) in 384-wells plates (confirmation I) and then in transwell plates (confirmation II). Confirmed hits were subjected to further validation using secondary assays.

Orthogonal assays

Western Blot based phagocytosis assay:

To distinguish total and internalized POS by means of western blot, half of the samples were treated with 2 mM EDTA for 10 minutes to remove bound POS from the cells before lysis and the other half was left with PBS as described previously (Mao and Finnemann, 2013). Around one million cells were lysed with RIPA buffer for 30 minutes at 4°C. Next, the protein lysates were separated from the pellet by centrifugation and around 50 µg of protein was loaded into Mini-PROTEAN® TGX™ Precast Protein Gels. 10 µl of Precision Plus Protein™ WesternC™ was loaded as a standard. Proteins were transferred into PVDF membrane using TE70 Semi-dry transfer unit. Blocking was done for one hour at room temperature in 5% milk dissolved in TBST. To analyze POS phagocytosis by means of western blot, membranes were incubated with mouse anti-RHO antibody (1:1000), which recognizes the C-terminal of the protein, followed by anti-rabbit β-TUBULIN (1:1000) antibody overnight at 4 °C in 5% milk-TBST. HRP-mouse secondary antibody (1:1000) and HRP-rabbit secondary antibody (1:1000) were added for one hour at room temperature in 5% milk-TBST. Precision Protein™ StrepTactin-HRP Conjugate was also added with the secondary to view the standard in chemiluminescence mode. The blot membrane was incubated with SuperSignal™ West according to Manufacturer´s instructions and imaged with LAS4000.

Fluorescence based phagocytosis assay in transwells

RPE cells grown in transwells were cut out from the well, transferred on slides and imaged using SP5-MP confocal microscope. During imaging thirty-one optical sections in three fields per well were acquired.

Trypan blue based phagocytosis assay

To distinguish bound and internalized POS by means of fluorescence imaging, 0.4% trypan blue or PBS were added to RPE cells in 384-wells plates for 10 minutes and then washed six times with PBS to remove leftover dye before methanol fixation at -20 ºC, as described previously (Almedawar et al., 2020; Mao and Finnemann, 2013). Trypan blue quenches bound POS in treated wells and allows the detection of internalized POS only. CV7000S confocal microscope was used for imaging.

FACS based phagocytosis assay:

To monitor the effect of RM on POS degradation AF555-labeled POS were added to the cells. After three hours the cells were washed and RM (20 µM) or Chloroquine (50 µM) were added to the cells for twenty-one more hours. Cells were then trypsinized and subjected to FACS sorting using Amnis® ImageStream®^{X} Mk II Imaging Flow Cytometer. Trypsinization detached bound POS allowing visualization of internalized POS as described in (Westenskow et al., 2012).

Scanning Electron Microscopy (SEM)

RPE cells on transwell filters were fixed with modified Karnovsky’s solution, containing 2% glutaraldehyde (GA) plus 2% PFA in 0.1 M phosphate buffer pH 7.4, until processing. For processing, samples were washed with PBS and post-fixed in 1% osmium tetroxide in PBS for two hours on ice. Next, they were washed with water and dehydrated in a graded series of ethanol starting from 30% and up to 100%. Then, they were critical-point dried using the Leica CPD 300 (Leica Microsystems, Vienna, Austria), cut out from the transwell, mounted on 12 mm aluminium stubs, and sputter-coated with gold using the Baltec SCD 050 (Leica Microsystems, Vienna, Austria). Finally, filters were analyzed with a Jeol JSM

7500F cold field emission SEM (Jeol, Eching, Germany) at 5 kV acceleration voltage using the lower secondary electron detector.

Transmission Electron Microscopy (TEM)

For immune-EM of POS seeded on RPE, the samples were fixed in 4% PFA. After several washes in PBS, the filters with the cells were dissected with a razor blade into small pieces (1-2 mm) and dehydrated and infiltrated in Lowicryl K4M resin using the progressive lowering of temperature (PLT) method (Carlemalm et al., 1982). After polymerisation, the blocks were raised up to room temperature, and 70 nm sections were cut on a Leica UC6 ultramicrotome. Sections were mounted onto copper mesh grids for immunolabeling. On-section labeling of ultrathin sections was performed as previously described (Fabig et al., 2012). In brief, sections were blocked with 1% BSA in PBS, incubated for one hour with primary antibodies mouse anti-RHO (1:200) in BSA/PBS. Next, they were washed with PBS, and incubated with bridging antibody (rabbit anti mouse, 1:100 in BSA/PBS) for thirty minutes. Sections were then washed with PBS, incubated with protein A gold (1:100) for one hour in BSA/PBS, washed with PBS, post-fixed in 1% glutaraldehyde/PBS, washed with water, stained with 4% uranyl acetate in water, washed with water and dried for TEM inspection. Ultrastructure and gold labeling was imaged with a FEI Morgagni 268D or a Jeol JEM1400 Plus both at 80 kV acceleration voltage.

Next-generation sequencing (RNA-seq)

HESC-RPE cultured in transwells were pretreated with either 20 μM RM or 0.2% DMSO in the presence of 0.3125 μg/ml MFGE8 for 1 hour. Then, POS were seeded on the cells and incubated for 3 hours before they were lysed and total RNA was purified from three consecutive RPE differentiation rounds, using Qiagen RNeasy Mini kit. Control samples included the same treatment without POS and untreated samples. Using the NEBNext Poly(A) mRNA Magnetic Isolation Module, polyadenylated mRNA was enriched from 1μg total RNA with an integrity number of ≥ 9, according to the manufacturer's instructions. The mRNA was eluted in 15 μl 2x first strand cDNA synthesis buffer (NEBNext), in order to chemically fragment the samples, followed by reverse transcription, second strand synthesis, end repair, A tailing and adapter ligation according to the manual of NEBNext Ultra Directional RNA Library Prep Kit for Illumina. For ligation, hybridized custom adaptors were used (Adaptor-Oligo 1 and 2). Afterwards, excess of non-ligated adapters were depleted by an XP bead purification, adding 1x bead. Finally, samples were indexed during a PCR enrichment step with 15 cycles of amplification using primers carrying a specific index sequence indicated with 'NNNNNN' (Index Primer1, 2 and 3). After two more XP beads purifications (0.9x) libraries were quantified using Fragment Analyzer Standard Kit. For Illumina flowcell production, samples were equimolarly pooled and then sequenced on two Illumina NextSeq flowcells in 75bp single-end mode.

Oligonucleotide sequence	SOURCE
Adaptor-Oligo 1: 5'-ACA CTC TTT CCC TAC ACG ACG CTC TTC CGA TCT 3'	This Study
Adaptor-Oligo 2: 5'-P-GAT CGG AAG AGC ACA CGT CTG AAC TCC AGT CAC-3'	This Study
Index Primer1: AAT GAT ACG GCG ACC ACC GAG ATC TAC ACT CTT TCC CTA CAC GAC GCT CTT CCG ATC T	This Study
Index Primer2: GTG ACT GGA GTT CAG ACG TGT GCT CTT CCG ATC T	This Study
Index Primer3: CAA GCA GAA GAC GGC ATA CGA GAT NNNNNN GTG ACT GGA GTT)	This Study

Resulting reads were mapped with GSNAP (v2017-08-15) to the human genome (hg38) using splice junction information from Ensembl (v81) as support. Uniquely mapped reads were then converted into counts per gene using featureCounts (v1.5.3) and gene annotations from Ensembl (v81). Normalization of the raw counts based on the library size and testing for differential expression between the different cell types/treatments was performed with the DESeq2 R package (1.18.1). Genes with adjusted p-value (Benjamini-Hochberg) less than 0.05 were considered differentially expressed. The heat maps presented in Figure 6 were generated using GraphPad Prism. Images in Figure S7 and data in Table S7 were generated using Ingenuity Pathway Analysis software (IPA).

Quantitative PCR (QPCR)

HESC-RPE cultured in transwells were pretreated with either 20 μM RM or 0.2% DMSO in the presence of 0.3125 μg/ml MFGE8 for 1 hour. Then, POS were seeded on the cells and incubated for 3 hours before they were lysed and total RNA was purified from three consecutive RPE differentiation rounds, using Qiagen RNeasy Mini kit. For reverse transcription, 500 ng of RNA were incubated with random primers and dNTP Mix for 5 minutes at 65 ºC. Next, SuperscriptII RT enzyme, 5X first strand buffer, DTT and RNAse out were added to the reaction. Primer hybridization, reverse transcription and enzyme deactivation were performed in the thermocycler according to the following program: 25 ºC for 10 minutes, 42 ºC for 50 minutes, 70 ºC for 15 minutes. For the qPCR 1.5 μl of the cDNA was added to the reaction containing SYBR™ Green PCR master mix S1PR5, POLR2A, MSFD2A and ERMN primers according to manufacturer's instructions. Quantitative PCR was performed on three biological experimental repeats and nine technical repeats, which include three cDNAs and from each cDNA three wells, per condition.

Transepithelial resistance (TER) measurement

TER was measured before treatment with RM and controls, and 3, 6, and 24 hours following treatment with the EVOM^2 using STX2 electrodes. Represented TER values were calculated as follows:

$$TER\ (\Omega * cm^2) = \left(Sample\ TER\ (\Omega) - Blank\ TER\ (\Omega)\right) \times\ Area\ cm^2$$

Whereby, blank is a transwell coated with GFR matrigel, and contains the same amount of media as the sample, and the area of the used transwells is 0.03 cm^2.

VEGF ELISA based Measurement

Media from the upper chamber and the lower chamber of the transwells where RPE cells were cultured was collected and frozen at -80 ºC before treatment and twenty-four hours after treatment with RM and controls. Media was diluted 1:5, and VEGF ELISA was performed according to manufacturer's instructions.

References

Almedawar, S., Vafia, K., Schreiter, S., Neumann, K., Khattak, S., Kurth, T., Ader, M., Karl, M.O., Tsang, S.H., Tanaka, E.M., 2020. MERTK-Dependent Ensheathment of Photoreceptor Outer Segments by Human Pluripotent Stem Cell-Derived Retinal Pigment Epithelium. Stem Cell Reports 14, 374–389.

Berthold, M.R., Cebron, N., Dill, F., Gabriel, T.R., Kötter, T., Meinl, T., Ohl, P., Thiel, K., Wiswedel, B., 2009. KNIME - the Konstanz information miner. ACM SIGKDD Explor. Newsl. 11, 26.

Carlemalm, E., Garavito, R.M., Villiger, W., 1982. Resin development for electron microscopy and an analysis of embedding at low temperature. J. Microsc. 126, 123–143.

Fabig, G., Kretschmar, S., Weiche, S., Eberle, D., Ader, M., Kurth, T., 2012. Labeling of Ultrathin Resin Sections for Correlative Light and Electron Microscopy. Methods Cell Biol. 111, 75–93.

Lamprecht, M.R., Sabatini, D.M., Carpenter, A.E., 2007. CellProfiler: Free, versatile software for automated biological image analysis. Biotechniques 42, 71–75.

Mao, Y., Finnemann, S.C., 2013. Analysis of Photoreceptor Outer Segment Phagocytosis by RPE Cells in Culture. Methods Mol. Biol. 935, 285–295.

Molday, R., Hicks, D., Molday, L., 1987. Peripherin . A Rim-Specific Membrane Protein of Rod Outer Segment Discs. Invest. Ophthalmol. Vis. Sci. 28, 50–61.

Westenskow, P.D., Moreno, S.K., Krohne, T.U., Kurihara, T., Zhu, S., 2012. Using Flow Cytometry to Compare the Dynamics of Photoreceptor Outer Segment Phagocytosis in iPS-Derived. Invest. Ophthalmol. Vis. Sci. 53, 6282–6290.

Zhu, Y., Carido, M., Meinhardt, A., Kurth, T., Karl, M.O., Ader, M., Tanaka, E.M., 2013. Three-Dimensional Neuroepithelial Culture from Human Embryonic Stem Cells and Its Use for Quantitative Conversion to Retinal Pigment Epithelium. PLoS One 8, e54552.

Zhu, Y., Schreiter, S., Tanaka, E.M., 2014. Accelerated Three-Dimensional Neuroepithelium Formation from Human Embryonic Stem Cells and Its Use for Quantitative Differentiation to Human Retinal Pigment Epithelium. Methods Mol. Biol. 1307, 345–355.
